# Experimental investigation of microstructural and performance evolution in ultrasonic-assisted stir-cast AA8011–5 wt.% TiB_2_ composites during multi-pass equal-channel angular pressing

**DOI:** 10.1371/journal.pone.0361373

**Published:** 2026-10-28

**Authors:** K. Chinna Maddaiah, G. B. Veeresh Kumar, Abhishek Agarwal, Balram Yelamasetti, N. Anuradha, K. Murali, Damcho Wangchuk

**Affiliations:** 1 Department of Mechanical Engineering, CMR Engineering College, Telangana, India; 2 Department of Mechanical Engineering, National Institute of Technology, Andhra Pradesh, India; 3 Department of Energy Technology, Tallinn University of Technology, Tallinn, Estonia; 4 Department of Mechanical Engineering, Sreyas Institute of Engineering and Technology, Hyderabad, Telangana, India; 5 Department of Mechanical Engineering, MLR Institute of Technology, Telangana, India; 6 Department of Civil Engineering, Marri Laxman Reddy Institute of Technology and Management, Hyderabad, India; 7 Department of Mechanical Engineering, College of Science & technology, Royal University of Bhutan, Phuentsholing, Bhutan; King Mongkut's University of Technology North Bangkok, THAILAND

## Abstract

The development of aluminium matrix composites with improved structural and tribological performance has attracted considerable interest for lightweight engineering applications. This work investigates the microstructural, mechanical, tribological, and hot-deformation response of an AA8011 aluminium composite containing 5 wt.% TiB_2_, fabricated by ultrasonic-assisted stir casting and subsequently processed by equal-channel angular pressing (ECAP). The effect of accumulated severe plastic deformation was evaluated in the as-cast condition and after 2, 4, 6, 8, and 10 ECAP passes. Phase constitution and elemental characteristics were examined using X-ray diffraction and energy-dispersive spectroscopy, while microstructural evolution was characterized using optical microscopy, scanning electron microscopy, electron backscatter diffraction, and transmission electron microscopy. Repeated ECAP progressively refined the aluminium matrix, improved the spatial distribution of TiB_2_ particles, and reduced pronounced casting-related structural discontinuities. The EBSD-measured grain size decreased from 12.45 μm in the as-cast condition to 0.54 μm after 10 passes. The resulting microstructural refinement and densification were accompanied by increases in hardness, tensile strength, compressive strength, flexural strength, impact energy, and elongation. The ultimate tensile strength increased from 182 to 266 MPa after 10 passes. Dry-sliding tests performed at loads of 10–50 N and a sliding distance of 5000 m showed reductions in wear rate of approximately 55–58% and in coefficient of friction of approximately 62–75% relative to the as-cast condition. Processing-map analysis further indicated an expansion of the stable deformation region with increasing ECAP passes. The combined results demonstrate that ultrasonic-assisted stir casting followed by multi-pass ECAP provides an effective route for tailoring the microstructure and mechanical, tribological, and hot-deformation response of AA8011−5 wt.% TiB_2_ composites within the investigated processing conditions.

## Introduction

Lightweight structural materials have attracted considerable attention because of the increasing demand for fuel-efficient systems, reduced emissions, and reliable performance under mechanical and tribological loading [1,2]. Aluminium and its alloys are widely used in transportation, automotive, aerospace, and general engineering applications because of their low density, good corrosion resistance, thermal conductivity, and ease of processing. Aluminium matrix composites (AMCs) provide an additional route for improving the mechanical and surface-related performance of aluminium while retaining the advantages associated with the aluminium matrix [3,4]. However, conventional aluminium alloys generally exhibit relatively low hardness and limited resistance to localized deformation and material removal under contact and sliding conditions [5]. The incorporation of hard ceramic reinforcements into the aluminium matrix can address these limitations by increasing load-bearing capability, restricting localized plastic deformation, and improving resistance to surface damage [6].

Several ceramic reinforcements, including SiC, TiC, Al_2_O_3_, and ZrO_2_, have been investigated for improving the strength, hardness, and wear resistance of aluminium-based composites [7]. The selection of a reinforcement depends not only on its hardness but also on its density, thermal stability, chemical compatibility, wettability, and ability to form a stable particle–matrix interface. Among these reinforcements, titanium diboride (TiB_2_) has received considerable interest because of its high hardness, high thermal stability, relatively low density, and favourable compatibility with aluminium [8]. TiB_2_ can also influence the solidification structure of aluminium when appropriate particle–melt interfacial conditions are established. During solidification, the presence of TiB_2_ may promote heterogeneous nucleation and modify the development of the α-Al matrix structure [9]. The resulting microstructural changes can contribute to improvements in hardness, strength, and resistance to material removal. Nevertheless, the effectiveness of TiB_2_ reinforcement depends strongly on its incorporation and spatial distribution within the aluminium matrix. Particle clustering, inadequate wetting, entrapped gases, and residual porosity can introduce local heterogeneity and weaken the particle–matrix interface, thereby limiting the expected benefits of the reinforcement [10].

Stir casting is one of the most commonly used liquid-metallurgy techniques for producing aluminium matrix composites because of its relatively simple processing route, scalability, and comparatively low manufacturing cost. However, conventional stir casting does not always provide adequate particle dispersion, particularly when the reinforcement has limited wettability with the molten aluminium. Inadequate incorporation may result in particle-rich regions, entrapped gases, interfacial defects, and residual porosity. These features can adversely affect both mechanical integrity and tribological behaviour. Consequently, several modified liquid-processing approaches have been investigated to improve reinforcement incorporation, melt circulation, particle dispersion, and casting quality.

Among these approaches, ultrasonic-assisted stir casting has shown considerable potential for improving the incorporation of ceramic particles into molten aluminium. The application of ultrasonic energy generates cavitation within the melt, and the subsequent collapse of cavitation bubbles produces localized pressure pulses and intense melt agitation. These effects can assist in breaking up particle agglomerates and improving the incorporation of reinforcement particles into the molten metal [11]. Acoustic streaming generated during ultrasonic treatment can further enhance melt circulation and facilitate particle redistribution throughout the liquid matrix [12]. As a result, ultrasonic treatment can contribute to improved particle dispersion, reduced casting-related defects, and stronger particle–matrix interaction [13]. Such improvements are particularly relevant for TiB_2_-reinforced aluminium composites, where the initial quality of the cast microstructure can strongly influence the response to subsequent deformation processing.

Although ultrasonic treatment can improve the initial cast condition, further microstructural refinement may be required to obtain a combination of high strength, hardness, ductility, and improved surface performance. Severe plastic deformation (SPD) techniques have therefore been widely investigated as post-processing methods for producing refined-grained aluminium alloys and composites. Equal-channel angular pressing (ECAP) is particularly attractive because it introduces substantial plastic strain through simple shear while maintaining the approximate cross-sectional dimensions of the processed billet, allowing repeated passes to be applied to the same material [14]. During successive ECAP passes, the accumulated strain promotes grain subdivision, dislocation accumulation and rearrangement, development of subgrain structures, and progressive evolution of low-angle and high-angle grain boundaries [15–17]. These microstructural changes can produce ultrafine-grained structures and consequently modify the mechanical response of aluminium-based materials.

Repeated ECAP deformation can also modify the spatial arrangement of reinforcement particles and the integrity of the cast structure. Severe shear deformation can redistribute localized particle-rich regions, reduce the influence of casting-related discontinuities, and improve the continuity of the particle–matrix interface [18]. The resulting changes are relevant to tribological behaviour because grain refinement increases the resistance of the aluminium matrix to localized plastic deformation, while a more uniform reinforcement distribution can provide improved support against contact loading. A refined and consolidated microstructure may therefore reduce the extent of material detachment and surface damage during sliding. The development of a relatively compact and stable surface layer can further influence the wear response and coefficient of friction [19,20]. However, the tribological behaviour of an ECAP-processed aluminium composite remains dependent on the applied load, counterface, sliding distance, surface condition, and other test parameters; therefore, improvements observed under one set of conditions should be interpreted within the corresponding experimental range.

In addition to room-temperature mechanical and tribological performance, understanding the hot-deformation behaviour of aluminium matrix composites is important because thermomechanical processing involves the simultaneous effects of temperature, strain, and strain rate. The Dynamic Materials Model (DMM) provides a framework for evaluating the relationship between material response and processing variables and for constructing processing maps based on power-dissipation efficiency and deformation stability [21]. Such maps can be used to identify regions associated with different deformation responses and to distinguish experimentally investigated domains in which stable or unstable deformation behaviour may occur. Processing-map analysis has therefore been applied to aluminium alloys and metal-matrix composites to assist in the development of thermomechanical processing schedules [22,23]. Nevertheless, DMM-derived parameters are dependent on the underlying flow-stress data and the procedure used to determine strain-rate sensitivity; consequently, the resulting maps need to be interpreted within the experimentally investigated temperature and strain-rate domain.

Despite the substantial literature concerning ultrasonic-assisted casting, TiB_2_-reinforced aluminium composites, ECAP processing, and hot-deformation behaviour, these aspects have generally been investigated as separate processing or property-related questions. The combined evolution of an ultrasonic-assisted stir-cast AA8011–5 wt.% TiB_2_ composite during successive ECAP passes, together with its microstructural, mechanical, tribological, fracture, and hot-deformation responses, remains insufficiently established [24–27]. In particular, a systematic pass-by-pass investigation is important because the initial microstructure produced during casting provides the starting condition for subsequent severe plastic deformation, while repeated ECAP progressively modifies grain structure, reinforcement distribution, porosity, and mechanical response. Establishing these relationships is also important for determining whether the improvements observed after ECAP arise from progressive grain refinement and structural consolidation, reinforcement redistribution, or their combined effects.

Accordingly, the present study investigates an ultrasonic-assisted stir-cast AA8011–5 wt.% TiB_2_ composite in the as-cast condition and after 2, 4, 6, 8, and 10 ECAP passes. The investigation considers the progressive evolution of grain structure, TiB_2_ distribution, structural integrity, mechanical properties, fracture characteristics, dry-sliding behaviour, and hot-deformation response. The experimental results are correlated to clarify the relationship between accumulated ECAP strain, microstructural evolution, and the resulting material performance. Particular attention is given to the contribution of grain refinement, deformation-induced dislocation strengthening, particle–matrix constraint, load transfer, and structural consolidation. The role of TiB_2_ is discussed in relation to particle–matrix interaction and deformation behaviour, while avoiding the assumption that the relatively coarse starting reinforcement directly pins the submicrometre grain boundaries generated during severe plastic deformation.

### Study rationale and contribution

The specific contribution of the present study is the systematic pass-by-pass linkage between processing, microstructure, mechanical performance, tribological behaviour, and hot-deformation stability in ultrasonic-assisted stir-cast AA8011−5 wt.% TiB_2_. The composite was examined in the as-cast condition and after 2, 4, 6, 8, and 10 ECAP passes, allowing the progressive effect of accumulated severe plastic deformation to be followed rather than inferred from selected processing conditions. The investigation combines XRD, EDS, optical microscopy, SEM, EBSD, TEM, mechanical testing, tensile-fracture analysis, dry-sliding wear evaluation, worn-surface characterization, and Dynamic Materials Model-based processing maps. This combination provides independent evidence linking grain refinement and TiB_2_ redistribution with changes in strength, ductility, wear response, and hot-deformation stability. The strengthening response is interpreted using grain-boundary strengthening, deformation-induced dislocation strengthening, load transfer, and particle-induced constraint, while the experimental evidence is considered collectively rather than assigning the measured improvement to a single mechanism. The resulting processing–microstructure–property relationship provides a more complete assessment of the investigated AA8011−5 wt.% TiB_2_ system than evaluations restricted to either casting, ECAP, mechanical properties, or tribological behaviour alone.

## Materials and methods

### Materials

The experimental work used AA8011 aluminium alloy (Al-Fe-Si alloy system) as the matrix material. This alloy was selected because of its favourable combination of low density, corrosion resistance, formability, and mechanical stability that makes it suitable for lightweight engineering applications. The alloy was procured from Bharat Aerospace Metals Ltd., Mumbai, India. Titanium diboride (TiB_2_) particles with an average particle size of approximately 50 μm were selected as the reinforcing material to improve the strength and wear resistance of the aluminium matrix. The reinforcement powder was supplied by Sigma-Aldrich and possessed high chemical purity together with a controlled particle size distribution. The use of a high-quality reinforcement was intended to achieve uniform particle incorporation during casting, improve the integrity of the particle-matrix interface, and produce a homogeneous microstructure. These characteristics provided a controlled reinforcement condition for the subsequent casting and ECAP experiments.

### Fabrication of AA8011–5 wt.% TiB_2_ composite

The AA8011−5 wt.% TiB_2_ composite was fabricated by a combination of mechanical stirring and ultrasonic melt treatment to improve the particle incorporation and casting uniformity. The AA8011 alloy was first heated to about 800 °C in an electric resistance furnace to obtain a fully molten charge. The melt temperature was then lowered to about 750 °C and held at this temperature during the addition of the reinforcement. A graphite-coated impeller rotating at 350 rev/min was employed to create a stable vortex in the liquid aluminium. Then 0.5 wt.% Mg was added to the melt to improve TiB_2_ wettability and promote more effective particle–matrix contact [28]. A relatively low Mg addition was selected to facilitate particle incorporation while avoiding the introduction of a large additional alloying contribution that could complicate interpretation of the subsequent ECAP response. The Mg addition was therefore kept constant for all processing conditions and was not treated as an experimental variable. Before addition, TiB_2_ particles were pre-heated to remove surface moisture and minimize gas-related defects. The nominal average TiB_2_ particle size specified for the starting reinforcement powder was approximately 50 μm. The particle dimensions visible in individual SEM fields after casting and ECAP were not used to redefine the starting powder specification because deformation, sectioning, particle fragmentation, and two-dimensional projection can produce substantially smaller apparent dimensions. The hot powder was progressively added in the vortex, and stirring was continued for 10 min to favor particle distribution throughout the melt [29]. The resulting slurry was then ultrasonically treated for a further 10 min using a 20 kHz unit at 2 kW. Cavitation and acoustic streaming induced during sonication promoted melt circulation, separated loosely bonded particle clusters, and enhanced contact between the reinforcement and aluminium matrix. These effects promoted a finer and more homogeneous solidification structure and reduced the likelihood of particle agglomeration and casting-related defects [30,31]. The molten composite was poured into metallic moulds preheated to about 300 °C, following ultrasonic treatment, to reduce abrupt thermal gradients during solidification. The billets after casting were allowed to cool naturally in air and subsequently machined to the required dimensions for ECAP and specimen preparation. The composite contained a nominal 5 wt.% TiB_2_ reinforcement relative to the total composite mass. A 5 wt.% reinforcement level was selected based on the reported use of TiB_2_ contents in aluminium matrix composites and ECAP-processed TiB_2_-reinforced aluminium systems, including Al–5 wt.% TiB_2_ and AA6061–5 wt.% TiB_2_ compositions [20,32]. This concentration was adopted as a fixed reinforcement condition to permit the effect of accumulated ECAP deformation to be isolated without introducing reinforcement content as an additional experimental variable. The present study therefore does not establish an optimum TiB_2_ concentration; assessment of different reinforcement contents remains outside its scope. Standard metallic material testing procedures, including various mechanical, tribological, and high-temperature deformation tests, were performed for the AA8011−5 wt.% TiB2 composite. The tensile behavior was tested in accordance with ASTM E8/E8M-25 [33]. The compression tests were performed at room temperature in accordance with ASTM E9-19 [34]. The impact energy was determined according to the ASTM E23-25 standard, and the flexural behavior was determined using a three-point bending configuration with controlled specimen geometry, support span, and loading conditions [35]. Vickers hardness was calculated according to ASTM E92-23. The coefficient of friction and dry-sliding wear behavior were evaluated using a pin-on-disc tribometer as per ASTM G99-23 [36]. The deformation behavior at elevated temperatures was investigated using hot-compression tests according to ASTM E209-18 [37]. Hot-compression tests were conducted over the temperature range of 250–500 °C and strain-rate range of 10^−^⁴–10^2^ s ^−^ ^1^, consistent with the ranges used to construct the processing maps. A further analysis of the true stress–true strain data was conducted using the Dynamic Materials Model (DMM) under the adopted temperature and strain-rate conditions for the development of the processing maps and identification of favourable deformation regions and flow-instability-prone domains.

### ECAP processing and experimental characterization

Equal-channel angular pressing (ECAP) was carried out using a solid die with two intersecting channels, an internal channel angle (Φ) of 120° and an outer curvature angle (Ψ) of 22°, as shown in Fig 1. The specimens were processed through 2, 4, 6, 8, and 10 ECAP passes to progressively increase the imposed plastic strain. For the present ECAP die geometry, with an internal channel angle (Φ) of 120° and an outer curvature angle (Ψ) of 22°, the conventional analytical expression for equivalent strain gives an effective strain of approximately 0.63 per pass. Accordingly, the nominal accumulated equivalent strains are approximately 1.26, 2.53, 3.79, 5.06, and 6.32 after 2, 4, 6, 8, and 10 ECAP passes, respectively. These values are used only to describe the nominal accumulated ECAP strain associated with the processing schedule. The selected multi-pass schedule was used to systematically evaluate the progressive effects of accumulated severe plastic deformation on the microstructure and properties of the AA8011−5 wt.% TiB_2_ composite.

**Fig 1:**
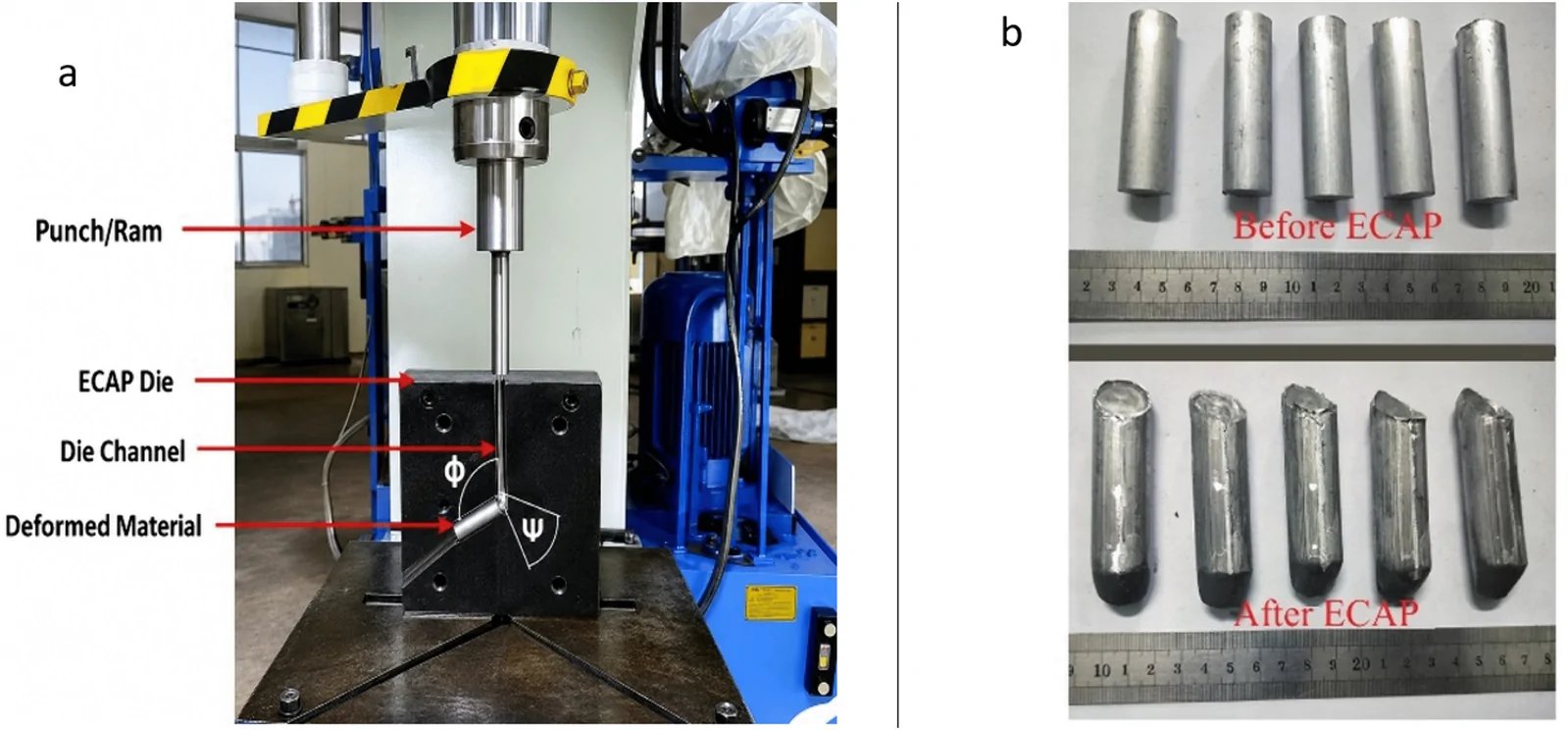
a) ECAP experimental setup b) Prepared samples.

Processing was carried out along route BC, in which the billet was rotated clockwise by 90° about its longitudinal axis between consecutive passes to promote a more uniform strain distribution and progressive grain subdivision. Molybdenum disulfide (MoS_2_) was used as a lubricant to reduce interfacial friction between the billet and die channels. The first ECAP pass was conducted at 350 °C to reduce the likelihood of surface cracking during the initial stage of severe plastic deformation. For the remaining passes, the processing temperature was decreased to 300 °C to maintain stable material flow while promoting further grain refinement [38]. Before each pressing operation, the billets were soaked at the selected temperature for 15 min to ensure uniform thermal conditions.

The effect of accumulated ECAP strain on the microstructure was systematically evaluated by characterizing the as-cast composite and specimens processed through 2, 4, 6, 8, and 10 ECAP passes. X-ray diffraction was used to identify the constituent crystalline phases and deformation-related changes in crystallographic behaviour. Grain morphology, TiB_2_ distribution, pore evolution, and progressive homogenization of the composite structure were examined using optical microscopy and scanning electron microscopy. The elemental constituents and spatial distribution of TiB_2_ within the aluminium matrix were further examined using energy-dispersive spectroscopy [20,39]. EBSD measurements were performed on polished sections of the AA8011–5 wt.% TiB_2_ composite using a field-emission scanning electron microscope equipped with an EBSD detector. The EBSD data were acquired using a 0.5 μm step size over an analysed area of approximately 250 × 250 μm² for each processing condition. The acquired patterns were indexed using the instrument software, and data with poor indexing quality were removed during post-processing. Grain boundaries were identified using a 15° misorientation criterion, while grains containing fewer than 10 indexed pixels were excluded from the grain-size analysis. The reported mean grain sizes were determined from five EBSD fields for each processing condition, with the indexed grains within these fields used for the statistical grain-size assessment. KAM maps were generated using a first-neighbour kernel with a 5° maximum misorientation threshold, while the same acquisition and post-processing parameters were maintained for all specimens to enable consistent comparison of deformation-induced lattice misorientation during successive ECAP passes. Tensile-fracture surfaces were examined by SEM to identify characteristic features including dimples, cleavage-like facets, microvoid coalescence, cracking, and particle–matrix separation. Tribological tests were also conducted under controlled loading and sliding conditions to evaluate the influence of accumulated ECAP strain on the dry-sliding performance of the AA8011–TiB_2_ composite through measurements of wear rate and coefficient of friction.

### Processing map analysis

Processing maps describe the relationship between the deformation temperature, strain rate, and the resulting flow behavior of a material. The energy put in during forming is assumed in the Dynamic Materials Model to be used up in plastic flow and microstructural rearrangement. This approach allows favourable deformation conditions to be separated from domains in which flow localization or damage may occur. For an aluminium-5 wt% TiB_2_ composite processed by equal-channel angular pressing, the material response depends on matrix deformability, reinforcement dispersion and particle-matrix interfacial stability. A suitable combination of temperature and strain rate promotes uniform shear deformation, closes casting-induced pores and supports progressive grain subdivision. It can also improve the distribution of TiB_2_ particles throughout the aluminium matrix [40,41]. Conversely, unsuitable processing conditions may produce localized stresses, interfacial separation and microcrack development around the ceramic particles. Thus, a processing map provides a practical basis for identifying a stable hot-deformation processing window that supports microstructural uniformity, densification, and improved deformation reliability. The processing maps were developed using the Dynamic Materials Model (DMM). The strain-rate sensitivity was determined from the flow-stress data as


$$\begin{array}{c} \text{m }=\;\partial \text{ln}\sigma /\partial \text{ln}\dot{\epsilon } \end{array}$$ (1)

where σ is the flow stress and ε̇ is the strain rate. The power-dissipation efficiency was calculated as


$$\begin{array}{c} \eta \;=\text{ 2m}/(\text{m }+\;\text{1}) \end{array}$$ (2)

The flow-instability criterion was evaluated using the DMM instability parameter,


$$\begin{array}{c} \xi =\dot{\epsilon }\;\partial \text{ln}[\text{m}/(\text{m }+\;\text{1})]/\partial \text{ln}\dot{\epsilon }\;+\text{ m} \end{array}$$ (3)

where negative values of ξ indicate conditions susceptible to flow instability. The strain-rate sensitivity was obtained from the experimentally measured flow-stress data at constant temperature and strain. The derivatives used to calculate the strain-rate sensitivity and instability parameter were evaluated from the logarithmic flow-stress data within the experimentally measured strain-rate range. The processing maps were therefore interpreted only within the experimentally investigated temperature and strain-rate domain. Because the calculated power-dissipation efficiency depends directly on the strain-rate sensitivity, regions showing very high η values were interpreted together with the underlying flow-stress data and the numerical differentiation procedure. The resulting processing maps were used to distinguish stable and instability-prone combinations of deformation temperature and strain rate and to identify regions associated with relatively high power-dissipation efficiency.

## Results and discussion

### X-ray diffraction analysis

Fig 2(a–f) shows the XRD patterns of the AA8011–5 wt.% TiB_2_ composite in the as-cast condition and after 2, 4, 6, 8, and 10 ECAP passes. The diffraction peaks of all samples are attributed to the FCC α-Al matrix and hexagonal TiB_2_ reinforcement, indicating that the phase constitution of the composite remains unchanged after the applied processing route.

**Fig 2:**
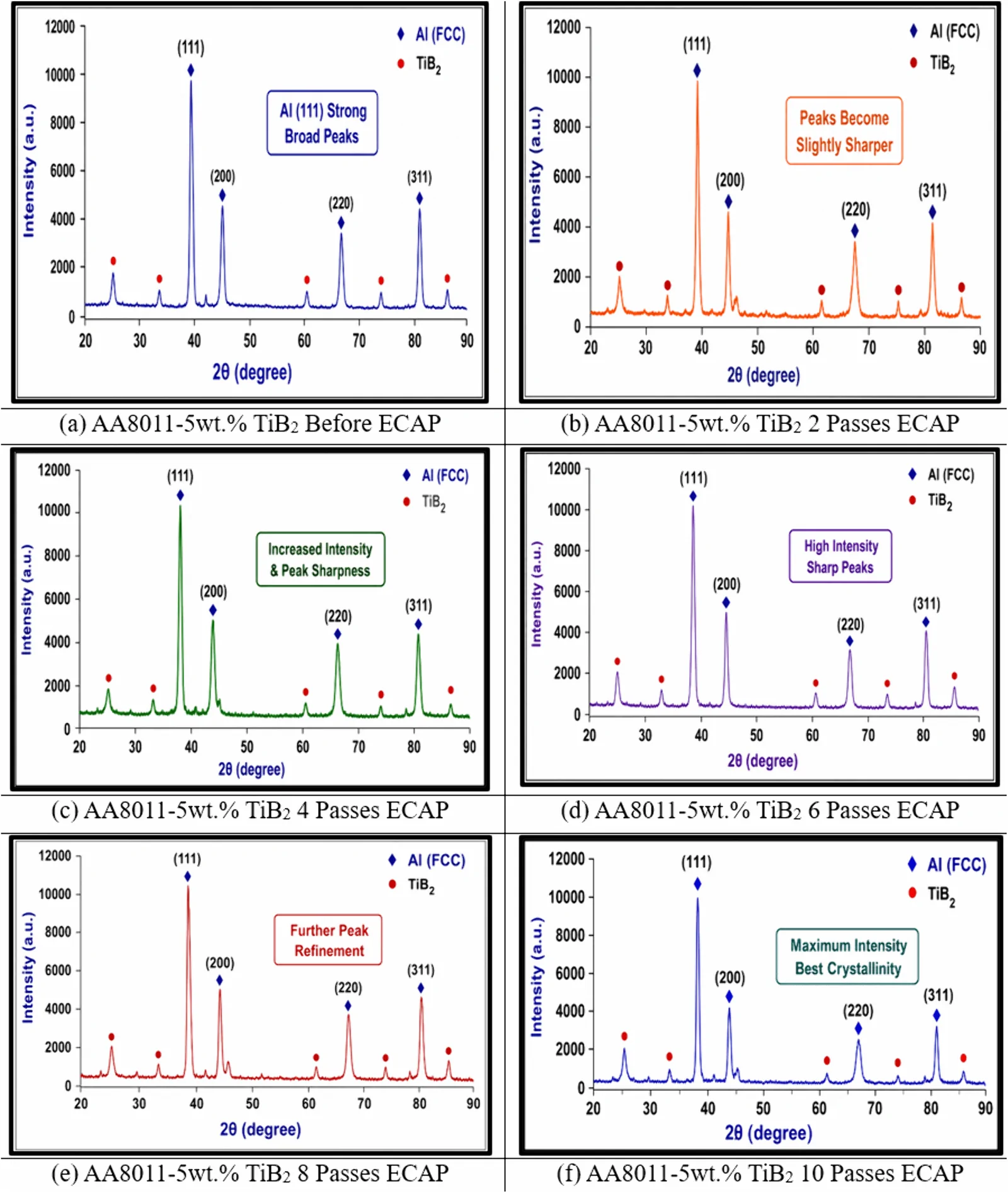
XRD patterns of the AA8011–5 wt.% TiB_2_ composite in the as-cast condition and after successive ECAP passes.

The diffraction maxima at 38.47°, 44.75°, 65.13°, and 78.17° correspond to the (111), (200), (220), and (311) planes of α-Al, whereas the peaks located at 27.37°, 35.98°, 41.76°, 51.42°, and 62.21° are attributed to the TiB_2_ phase. No other reflections were observed in the range of 2θ examined, indicating that no additional crystalline phase was detected within the resolution and scanned 2θ range of the XRD measurements. The intensity of the Al (111) diffraction peak increases progressively with increasing ECAP passes, with the measured peak intensity increasing from 8420 a.u. after 2 passes to 9150, 9870, 10450, 10980, and 11340 a.u. after 4, 6, 8, and 10 passes, respectively. The progressive change in peak intensity may be associated with changes in crystallographic texture or preferred orientation produced by repeated severe plastic deformation [42,43]. However, because pole figures or quantitative texture measurements were not obtained, the XRD peak-intensity variation is not taken as direct evidence of a specific texture component. However, peak intensity alone does not provide a direct measure of grain size or crystallite size. The imposed shear deformation promotes progressive grain subdivision, while the dispersed TiB_2_ particles can influence local deformation and dislocation interactions within the aluminium matrix. Given the relatively coarse starting particle size, the observed submicrometre grain refinement is attributed primarily to severe-plastic-deformation-induced grain subdivision and boundary evolution rather than direct grain-boundary pinning by TiB_2_. The absence of additional reflections indicates that no new crystalline phase was detected within the resolution of the XRD measurements. Accordingly, the XRD results indicate that the phase constitution remains stable during ECAP, while the ultrafine-grained structure observed after repeated processing is independently supported by the results presented in the sections on EBSD Analysis of Microstructural Refinement, Grain Size Evolution, and TEM Analysis of Microstructural Evolution. The maintained phase stability and progressive microstructural refinement are consistent with the observed increases in strength, hardness, and dry-sliding wear resistance after multi-pass ECAP.

### Energy-dispersive spectroscopy analysis

Fig 3(a–f) presents the EDS spectra obtained from the AA8011−5 wt.% TiB_2_ composite before ECAP and after 2, 4, 6, 8, and 10 ECAP passes. The spectra contain signals corresponding primarily to Al, Ti, B, O, and Fe, with no additional major elemental peaks appearing after ECAP. The Ti and B signals confirm the presence of the TiB_2_ reinforcement, while Al represents the matrix.

**Fig 3:**
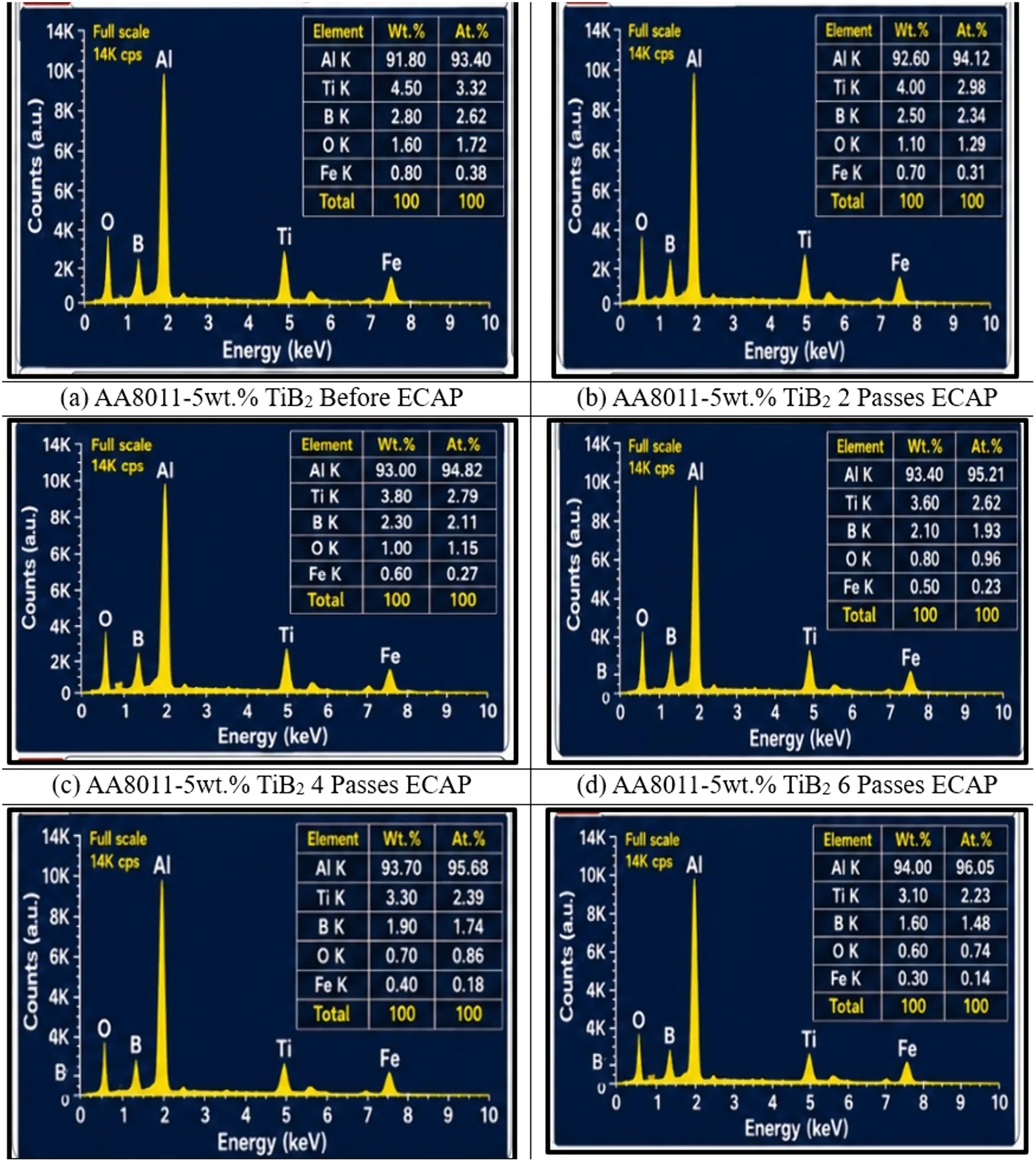
EDS spectra and semi-quantitative elemental composition of the AA8011–5 wt.% TiB_2_ composite in the as-cast condition and after successive ECAP passes.

The semi-quantitative EDS values vary gradually with ECAP pass number, with the measured Al signal increasing from 91.80 wt.% in the as-cast condition to approximately 94.00 wt.% after 10 passes, accompanied by decreases in the measured Ti, B, O, and Fe fractions. Because EDS provides a localized and semi-quantitative composition rather than a bulk chemical analysis, these variations are interpreted primarily as changes in the local sampling volume and elemental distribution associated with particle redistribution and microstructural homogenization, rather than as changes in the overall alloy chemistry. The absence of newly detected major elements further indicates that ECAP did not introduce an obvious new elemental constituent. Thus, the EDS results support the observed changes in local elemental distribution without being interpreted as evidence of a change in bulk composition [44,45].

### EBSD analysis of microstructural refinement

The EBSD inverse pole figure (IPF), grain boundary, and Kernel Average Misorientation (KAM) maps of the AA8011−5 wt.% TiB_2_ composite in the as-cast condition and after successive ECAP passes are shown in Fig 4(a–r). The successive ECAP processing led to a distinct change in the grain structure presented in the IPF maps. With increasing number of passes, the coarse grains in the initial condition were gradually subdivided into smaller and more uniformly distributed grains, confirming the efficiency of severe plastic deformation in promoting microstructural refinement. Grain boundary maps showed a parallel transition with continued processing, where the dominance of high-angle boundaries increases and the proportion of low-angle boundaries decreases.

**Fig 4:**
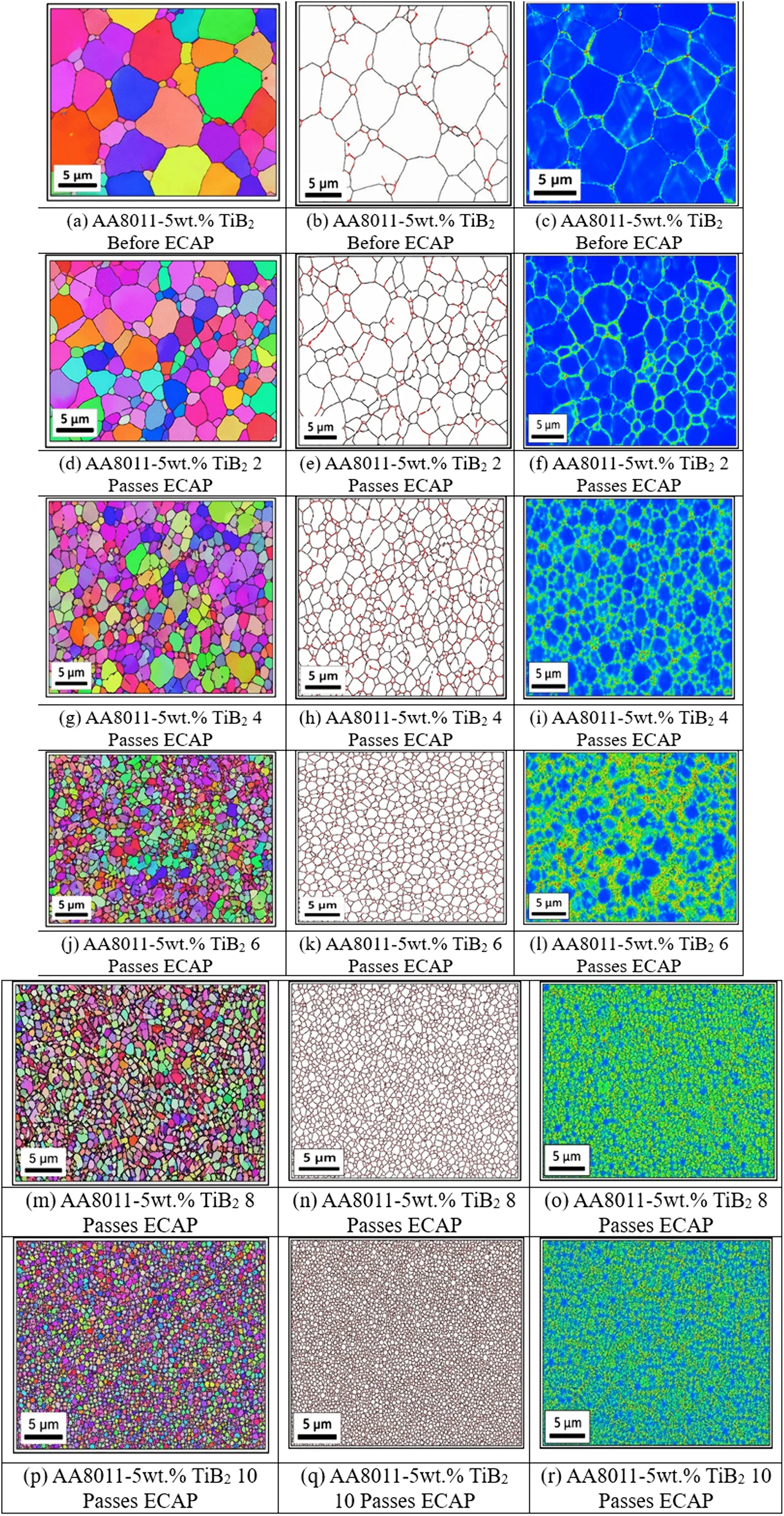
EBSD-derived inverse pole figure, grain-boundary, and kernel average misorientation maps of the AA8011–5 wt.% TiB_2_ composite in the as-cast condition and after successive ECAP passes.

This evolution indicates that the substructure generated by deformation is gradually converted into stable grain boundaries as ECAP processing continues. The KAM maps also show a redistribution of localized strain, with early passes creating regions of pronounced misorientation due to the rapid accumulation of dislocations under intense shear deformation. Subsequent passes lead to a more uniform misorientation pattern, indicating a homogeneous distribution of the plastic strain in the matrix. The progressive accumulation of intense shear deformation during ECAP drives the observed microstructural evolution by continuously reorganizing dislocations and transforming the developing subgrains into finer grain regions. Meanwhile, the dispersed TiB_2_ particles can contribute to resistance against local matrix deformation and dislocation motion; however, given the relatively coarse starting particle size, their direct role in pinning the submicrometre grain boundaries formed after severe deformation is not assumed here. Consequently, the specimen processed through 10 ECAP passes exhibits the most uniform grain morphology and the most stable boundary network among all processing conditions. The EBSD results therefore demonstrate that repeated ECAP effectively improves the microstructural stability and structural uniformity of the AA8011–5 wt.% TiB_2_ composite, which provides a consistent microstructural basis for the enhanced mechanical and tribological response observed after processing [46,47].

### Grain size evolution

Grain size study and the EBSD measurement outcomes show that ECAP has a crucial role in the changes of microstructure, as shown in Fig 5(a–f). The results indicate that grain size decreases continuously from about 12.45 μm in the initial (cast) state to 6.32, 3.18, 1.72, 0.96, and 0.54 μm after applying the ECAP technique for 2, 4, 6, 8, and 10 passes, respectively. The simultaneous reduction in mean grain size and increase in the number of detected grains provides quantitative evidence that the progressive structural refinement is not limited to local morphological changes observed in individual micrographs.

**Fig 5:**
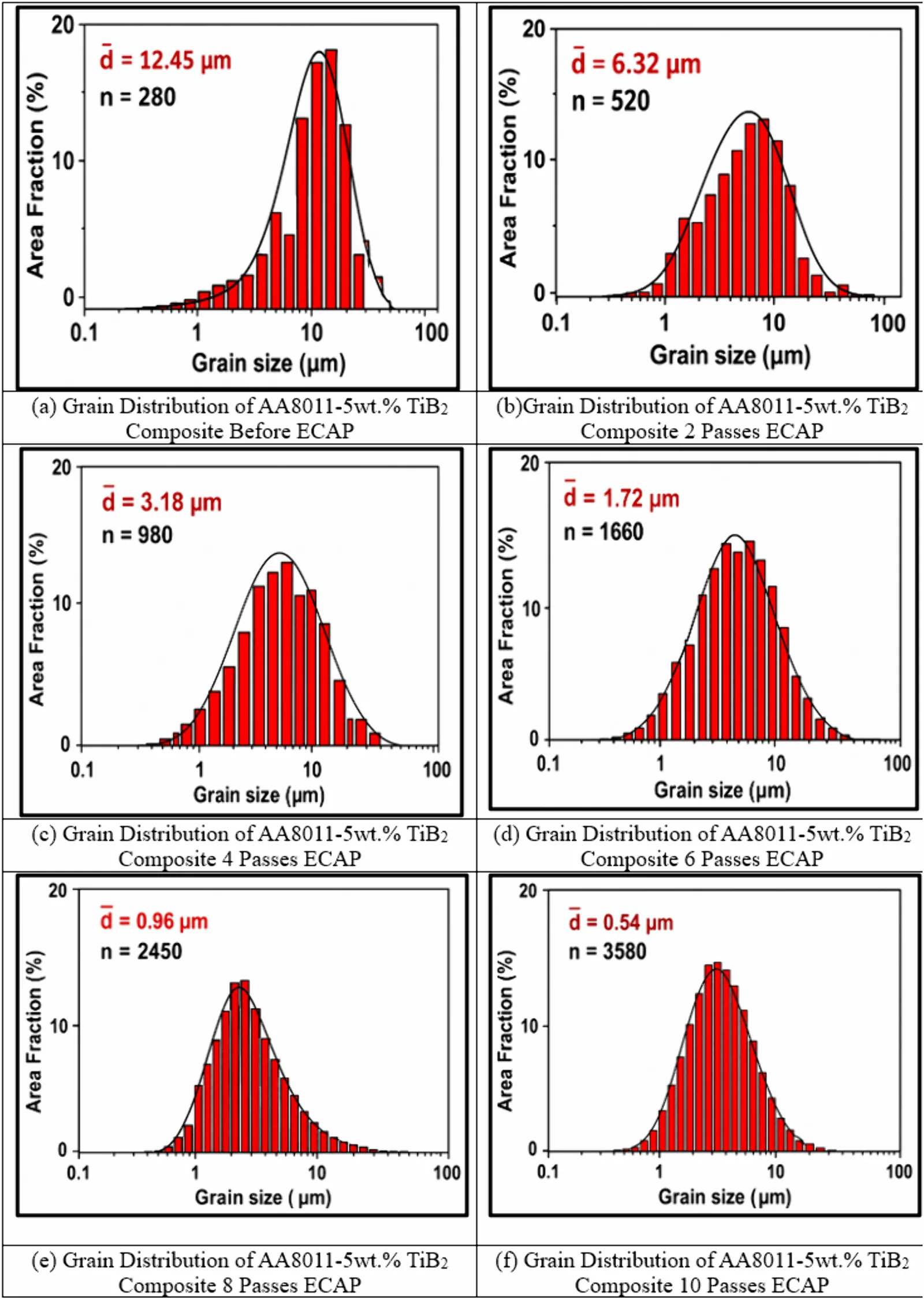
Grain-size distributions of the AA8011–5 wt.% TiB_2_ composite in the as-cast condition and after successive ECAP passes.

A noticeable shift of the distribution towards the finer grain-size range is also observed with increasing ECAP passes, indicating that coarse grains are progressively replaced by uniformly distributed ultrafine grains. This behaviour is attributed to the repeated shear deformation imposed during ECAP, which continuously fragments the original grains and promotes the formation of new grain boundaries. The TiB_2_ particles present can additionally influence local strain partitioning and dislocation accumulation during deformation. The progressive reduction in grain size observed here is therefore attributed primarily to severe-plastic-deformation-induced grain subdivision and boundary evolution, with the reinforcement contributing to the deformation response rather than being assumed to directly pin the submicrometre grain boundaries. Hence, the specimen deformed by 10 ECAP passes shows the finest and most homogeneous grain structure compared to all processing conditions. The measured grain-size evolution provides a quantitative basis for considering the grain-refinement contribution to strengthening [48]. According to the Hall–Petch relationship, σ_γ_ = σ₀ + k_γ_d ^−^ ^1/2^, the reduction in mean grain size from 12.45 μm to 0.54 μm increases the grain-boundary strengthening term proportional to d ^−^ ^1/2^. Related composite studies have also shown that reinforcement morphology, interfacial characteristics, constituent distribution, and micromechanical load transfer can strongly influence the resulting mechanical response, although the specific mechanisms depend on the matrix and reinforcement system [49–51]. However, a unique numerical strengthening contribution cannot be determined without independently establishing the Hall–Petch coefficient for the present AA8011–TiB_2_ system; therefore, the measured strength increase is interpreted as the combined effect of grain refinement, deformation-induced dislocation strengthening, particle–matrix constraint, load transfer, and structural consolidation.

### Optical microstructural evolution

Fig 6(a–f) shows the optical microstructural evolution of the AA8011–5 wt.% TiB_2_ composite after successive ECAP passes. The original microstructure consists of relatively coarse equiaxed grains and some isolated TiB_2_-rich regions, which is consistent with the normal solidification behavior of the cast composite. After 2 ECAP passes, the grains are distorted and aligned along the direction of material flow, confirming the introduction of substantial shear deformation. This deformation reduces the extent of particle clustering and redistributes the TiB_2_ particles more homogeneously [52]. The elongated grains are progressively subdivided into smaller structural units with further processing to 4 ECAP passes, leading to a finer and more uniform microstructure. The distribution and microstructure regularity of the reinforcement particles in the aluminium matrix are improved.

**Fig 6:**
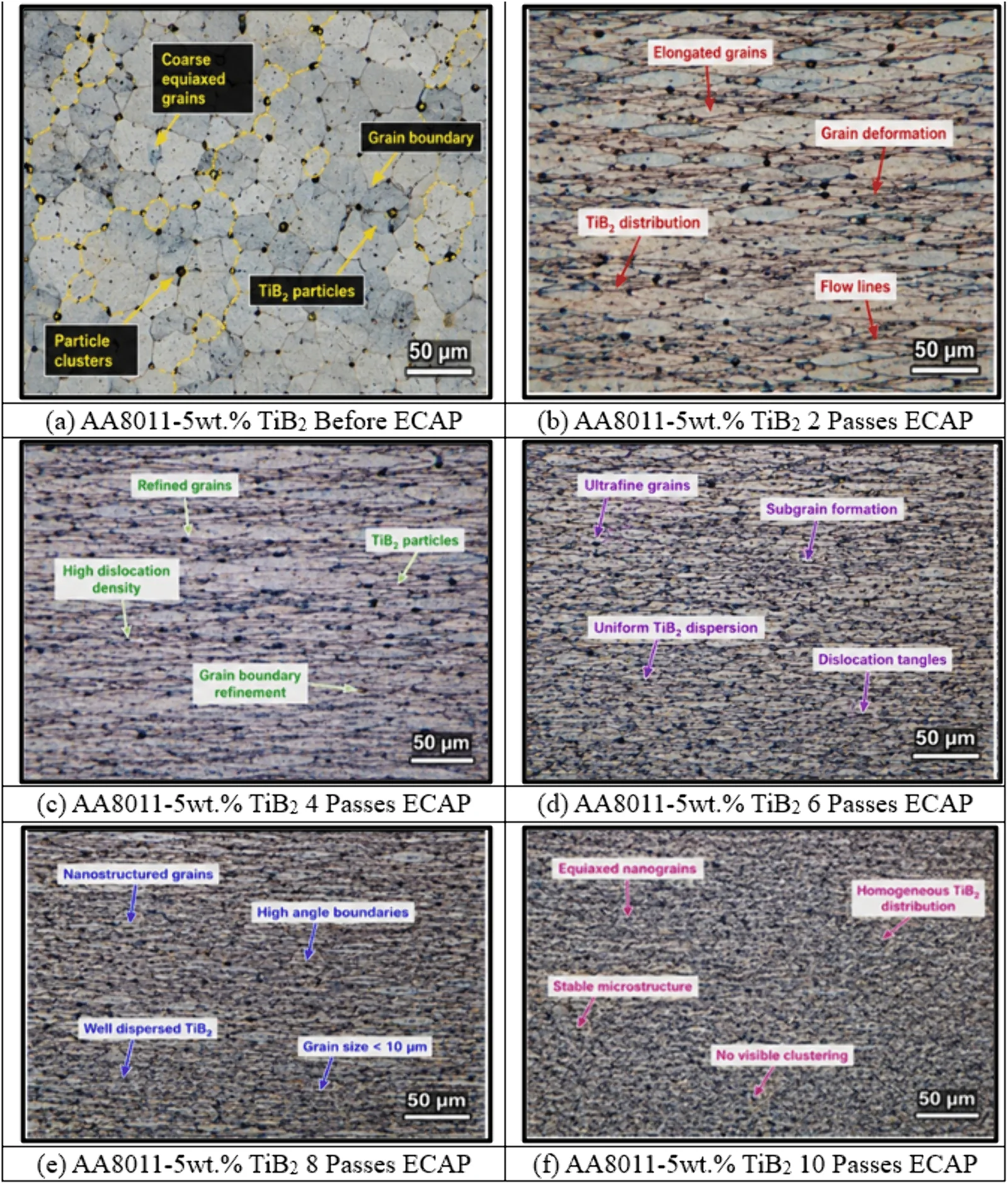
Optical micrographs of the AA8011–5 wt.% TiB_2_ composite showing grain restructuring and TiB_2_ redistribution during successive ECAP passes.

The grain size reduction is more obvious at 6 ECAP passes, and the microstructure consists mainly of ultrafine grains with uniform distribution of particles. Further deformation to 8 and 10 ECAP passes leads to a dense network of fine equiaxed grains in the observation area. No obvious agglomeration of particles is detected under these conditions, indicating that the repeated ECAP can effectively keep a stable dispersion of the TiB_2_ reinforcement. The microstructural evolution observed can be attributed to the accumulation of plastic strain with successive ECAP passes that continuously subdivide the original grains, while the TiB_2_ particles influence local deformation through particle–matrix constraint and dislocation interaction. The formation of a homogeneous ultra-fine-grained microstructure after multiple ECAP passes provides a strong structural basis for enhancing the mechanical and tribological properties of the AA8011−5 wt.% TiB_2_ composite [53].

### SEM analysis of TiB_2_ distribution and grain refinement

Fig 7(a–f) shows the SEM micrographs of the AA8011−5 wt.% TiB_2_ composite before and after the ECAP process. The as-cast material exhibited a relatively coarse aluminium grain structure and isolated zones with a high concentration of TiB_2_ particles. Limited numbers of microvoids were also seen, and this may indicate that complete structural uniformity was not achieved during solidification. After two ECAP passes, the aluminium matrix underwent intense plastic strain, which led to the elongation and preferential orientation of the original grains in the direction of the applied shear. The applied deformation also redistributes the reinforcement particles more efficiently, leading to a clear reduction of the particle-rich clusters. The original grain morphology is gradually broken down into finer structural units with increasing deformation to 4 ECAP passes, resulting in a more compact, uniform microstructure. The TiB_2_ particles appear more uniformly distributed in the examined matrix regions, with less pronounced spatial variation in interparticle spacing than in the initial condition. The microstructure is composed of ultrafine grains approaching the submicrometre scale after 6 ECAP passes, with a dense and continuous grain boundary network resulting from extensive grain subdivision.

**Fig 7:**
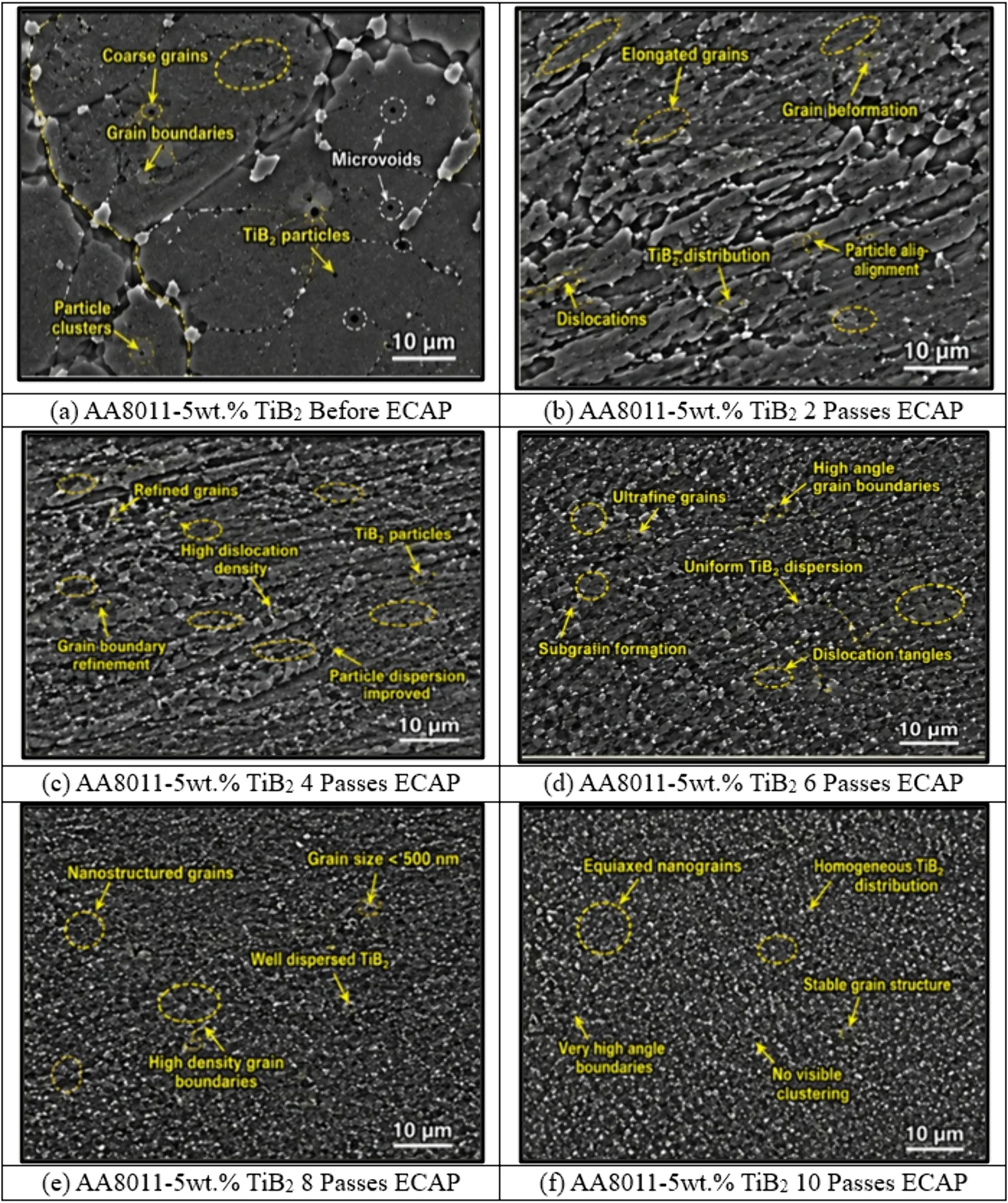
SEM micrographs of the AA8011–5 wt.% TiB_2_ composite showing grain refinement and redistribution of TiB_2_ particles during successive ECAP passes.

The reinforcement appears more uniformly distributed, and no pronounced particle-rich clustering is evident within the examined SEM fields. These observations are based on the examined two-dimensional micrographs; a formal statistical clustering or nearest-neighbour analysis was not performed. Further processing to 8 and 10 ECAP passes results in a more homogeneous microstructure with fine equiaxed grains and well-dispersed TiB_2_ particles within the examined regions. The pronounced voids and reinforcement-rich regions observed in the initial cast composite were reduced in the examined SEM fields after repeated deformation passes, indicating a denser and more homogeneous local microstructure. During equal-channel angular pressing, the repeated application of intense shear deformation progressively broke down the initially coarse grains into much smaller crystallographic regions. The TiB_2_ particles can interact with dislocations and contribute to local strain partitioning during severe deformation. The refined structure observed after repeated ECAP is therefore attributed primarily to progressive grain subdivision and boundary evolution, while particle–matrix interactions contributed to the deformation response. The refined and more uniformly distributed microstructure observed after 10 ECAP passes provides favourable conditions for improving mechanical response and wear resistance of the AA8011–5 wt.% TiB_2_ composite [54,55].

### Density and porosity evolution

Fig 8 Compares the theoretical density, measured density, and porosity of the AA8011 composite reinforced with 5 wt.% TiB_2_ in the as-cast state and after successive ECAP passes. The theoretical density of the AA8011–5 wt.% TiB_2_ composite was taken as 2.86 g cm ^−^ ^3^ and remained unchanged throughout ECAP because the nominal reinforcement fraction was constant. The experimental density was determined for each processing condition, and the corresponding porosity was calculated from the difference between the theoretical and measured densities using-.

**Fig 8:**
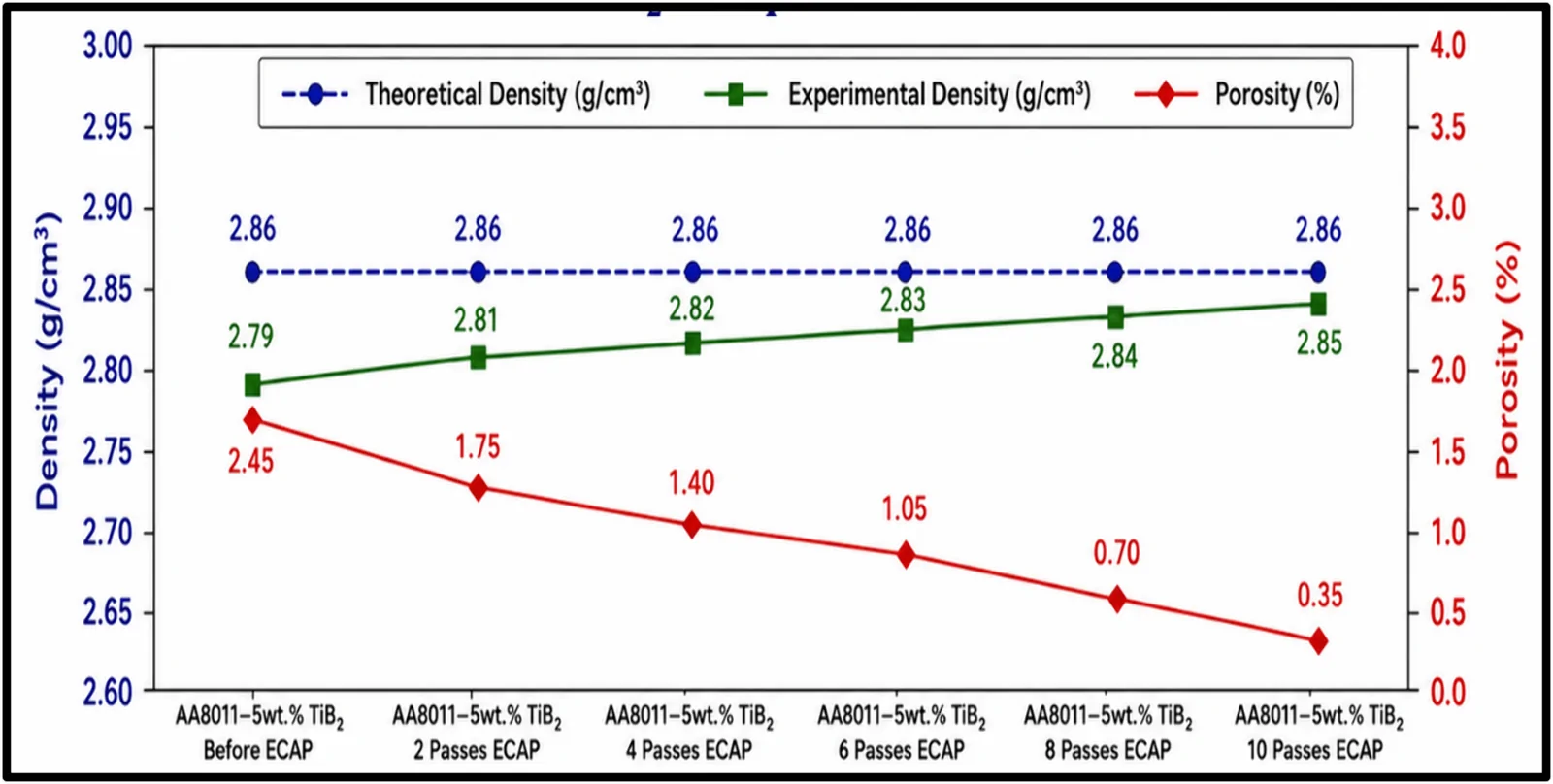
Theoretical and measured density and calculated porosity of the AA8011–5 wt.% TiB_2_ composite in the as-cast condition and after successive ECAP passes.


$$\begin{array}{c} \text{P }=\;[(\rho_{\text{th}}\;-\;\rho_{\text{exp}}/\;\rho_{\text{th}}]\;\times \text{ 100} \end{array}$$

where *P* is the porosity, ρ_th_ is the theoretical density, and ρ_exp_ is the experimentally measured density. For each processing condition, density measurements were repeated on independently prepared specimens, and the reported density was calculated from the repeated measurements. The measurement repeatability was assessed from the corresponding standard deviation. Because the porosity values were calculated indirectly from the measured density rather than determined by three-dimensional imaging, the reported porosity should be interpreted as an estimated bulk porosity fraction rather than a direct measurement of individual pore volume. The experimental density, however, increased progressively from 2.79 g cm ^−^ ^3^ before ECAP to 2.81, 2.82, 2.83, 2.84 and 2.85 g cm ^−^ ^3^ after 2, 4, 6, 8 and 10 passes, respectively. An opposite trend was observed for porosity.

The initial value of 2.45% decreased to 1.75% after two passes and continued to fall to 1.40%, 1.05%, 0.70% and 0.35% after 4, 6, 8 and 10 passes. The changes imply that the compactness of the composite was gradually increased with repeated ECAP. The improvement in density can be related to the closure and compression of internal pores under the intense shear deformation imposed during each pass. The repeated ECAP deformation promoted improved continuity of the aluminium matrix and a more uniform distribution of TiB_2_ particles within the composite. Such progressive homogenization diminished the local structural discontinuities and increased the effective contact area between the matrix and the reinforcement [56]. After 10 passes, the measured density was closest to the theoretical value, and the porosity was at its lowest level, indicating improved material consolidation and structural integrity. The progressive reduction in calculated porosity is consistent with improved material consolidation and structural integrity. These changes provide a plausible microstructural contribution to the improved mechanical and tribological responses observed after ECAP, while the processing-map results provide independent evidence of changes in hot-deformation stability.

### Mechanical property evolution

The mechanical response of the AA8011−5 wt.% TiB_2_ composite in the as-cast state and after 2, 4, 6, 8, and 10 ECAP passes is presented in Fig 9(a-f). For each property, an improvement was observed to proceed continuously with increasing accumulated deformation. Vickers hardness increased from 74 HV in the as-cast condition to 107 HV after 10 ECAP passes, while the ultimate tensile strength increased from 182 to 266 MPa. Similarly, the compressive strength also showed an increasing trend from 298 to 415 MPa and flexural strength from 248 to 352 MPa.

**Fig 9:**
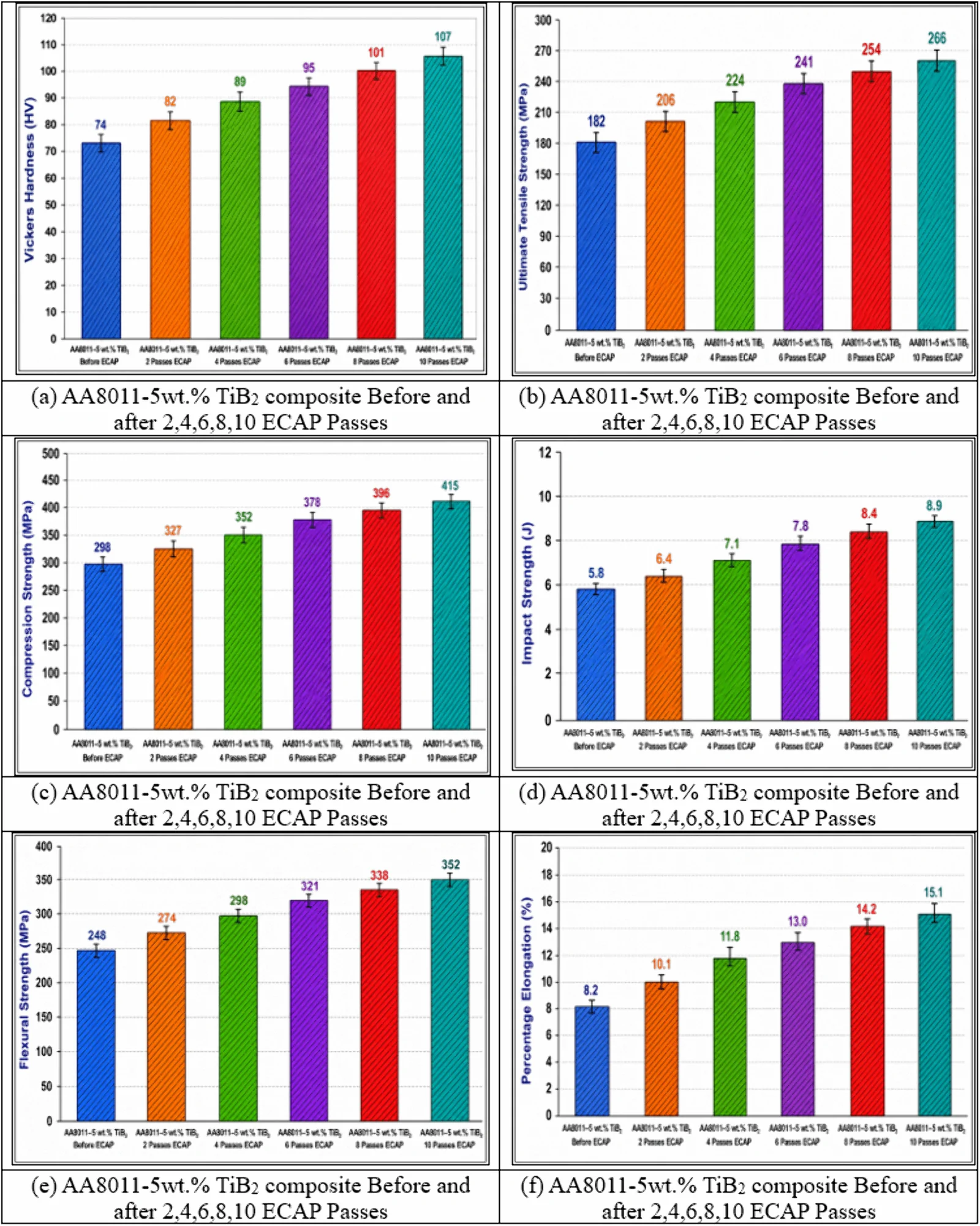
Mechanical properties of the AA8011–5 wt.% TiB_2_ composite in the as-cast condition and after successive ECAP passes.

Impact energy increased from 5.8 to 8.9 J. Also, the elongation increased from 8.2% in the initial condition to 15.1% after 10 passes. The concurrent enhancement of strength, impact energy, and elongation suggests that multiple ECAP processing improved the load-carrying capacity of the composite while retaining and even improving its capacity to undergo plastic deformation [32,57,58]. The progressive improvements in mechanical behaviour are closely connected with the microstructural changes induced during ECAP. Repeated shear deformation changes the coarse cast structure into a refined ultrafine-grained microstructure that provides a higher number of grain boundaries to oppose dislocation movement during deformation. The TiB_2_ reinforcement is, meanwhile, more homogeneously dispersed in the aluminium matrix, which enables a more efficient distribution of the applied load and reduces localized stress concentrations. The observed reduction in casting-related structural discontinuities and the more homogeneous TiB_2_ distribution further support the structural integrity of the composite by reducing preferential sites for crack initiation. The strengthening mechanisms are active at the same time with the increase of ECAP passes. The hardness, tensile strength, compressive strength, impact energy, and flexural strength, together with elongation, increased progressively with increasing ECAP passes. The specimen processed by 10 ECAP passes exhibited the highest measured values among the investigated processing conditions. Within the present experimental window, progressive ECAP was associated with simultaneous increases in strength-related properties, impact energy, and elongation. These observations describe the measured trends across the investigated processing conditions; no formal hypothesis-testing analysis was performed.

### Tensile-fracture morphology

Fig 10(a-f). Tensile-fracture surfaces of the AA8011−5 wt.% TiB_2_ composite in as-cast condition and after 2, 4, 6, 8 and 10 ECAP passes. The fracture surface of the unprocessed specimen consisted of wide dimples, cleavage-like facets, coalesced microvoids and areas showing detachment of TiB_2_ particles from the aluminium matrix.

**Fig 10:**
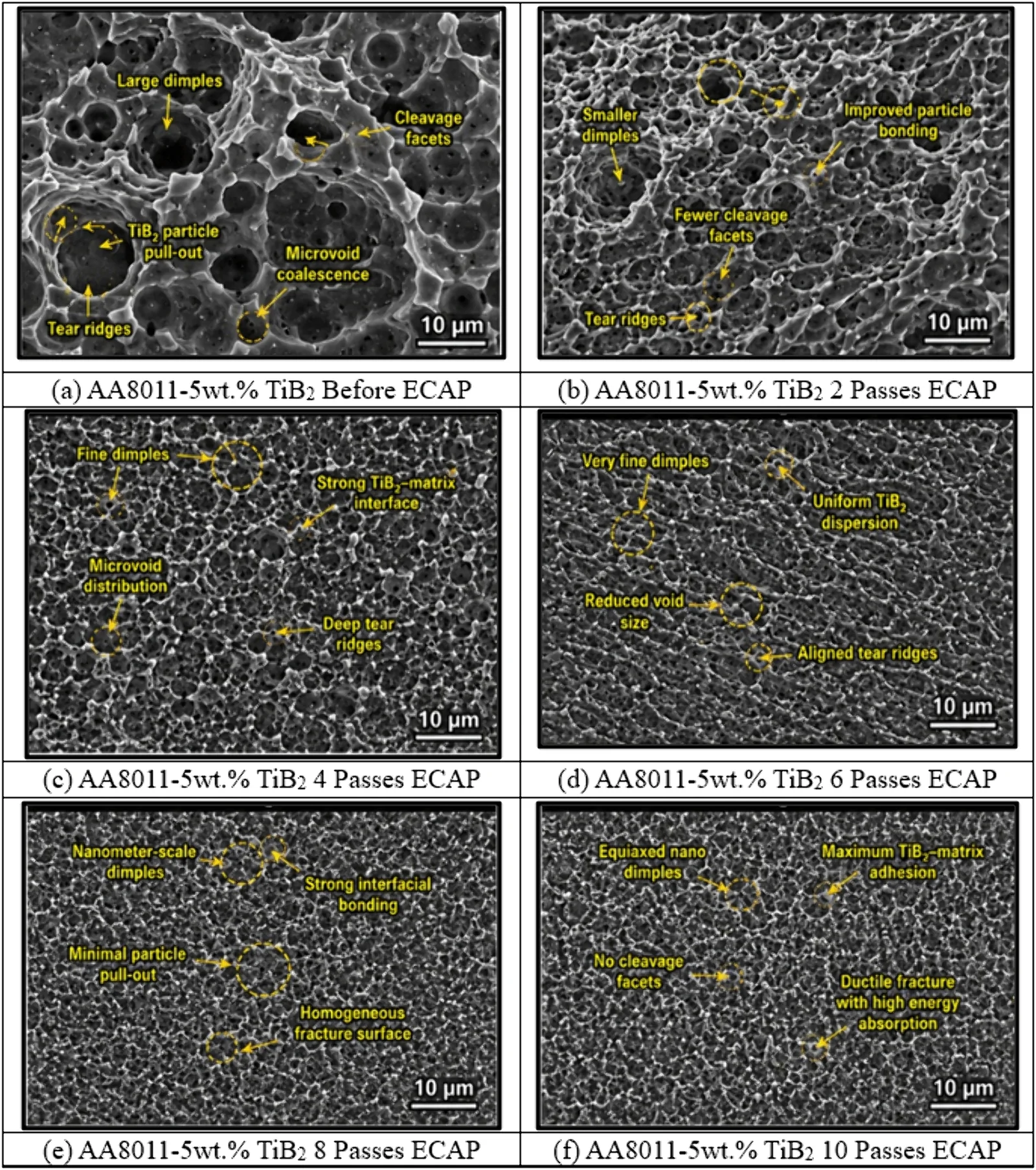
Tensile-fracture surfaces of the AA8011–5 wt.% TiB_2_ composite in the as-cast condition and after successive ECAP passes.

The coexistence of these features implies that failure occurred by a combination of ductile void growth and localized brittle cracking. Local stress raisers like regions with dense reinforcement and poor particle-matrix bonding may have contributed to the formation of voids and premature crack initiation during tensile loading. On the fracture surface after 2 ECAP passes, a finer dimple morphology and a marked reduction in cleavage areas can be observed. The reinforcement particles appear more effectively retained within the matrix, with fewer regions of apparent particle–matrix separation after deformation. Processing to higher levels (4 ECAP passes) leads to a more uniform fracture surface consisting of closely spaced fine dimples and well-developed tear ridges, which is indicative of a higher degree of plastic strain before fracture [59]. The fracture surface after six ECAP passes showed a more uniform aspect and a fine and uniformly distributed microvoid population with limited indications for TiB_2_ particle debonding from the aluminium matrix. Additional processing using 8 and 10 ECAP passes produces a dense network of fine equiaxed dimples across the examined fracture surfaces. Cleavage is not seen anymore, and particle pull-out is seldom observed, which indicates that failure is mostly by ductile microvoid nucleation and growth. The progressive refinement of the fracture morphology is related to the ultrafine-grained structure generated during ECAP, the improved distribution of TiB_2_ particles, and the reduction of casting-related defects. These microstructural changes are consistent with increased resistance to crack initiation and propagation within the examined fracture morphology, together with the measured increase in absorbed impact energy. Consequently, the specimen processed through 10 ECAP passes exhibits the most refined and predominantly ductile fracture morphology among the investigated conditions, consistent with the measured increase in tensile strength and elongation [60].

### TEM analysis of microstructural evolution

Fig 11(a–r) shows the TEM, SAED, and HRTEM observations of the AA8011–5 wt.% TiB_2_ composite in the as-cast condition and after successive ECAP passes. The as-cast material consists of relatively coarse α-Al grains with TiB_2_ particles dispersed within the matrix. After two ECAP passes, the grains become elongated along the principal deformation direction, accompanied by an increase in dislocation density.

**Fig 11:**
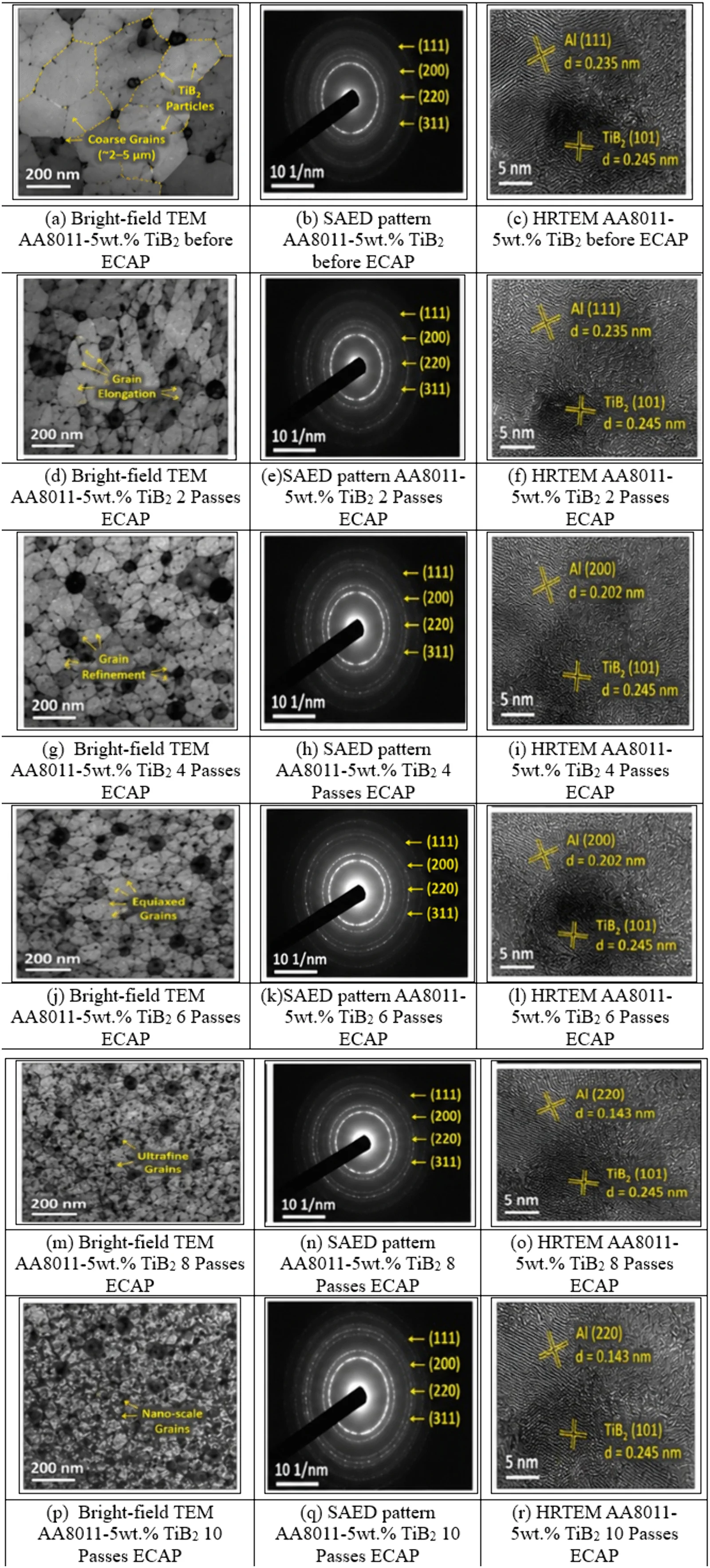
TEM, SAED, and HRTEM observations of the AA8011–5 wt.% TiB_2_ composite showing microstructural evolution from the as-cast condition through successive ECAP passes.

Further deformation during the fourth and sixth passes subdivides the elongated structures into smaller subgrains, which progressively evolve into finer equiaxed grains [61]. After the eighth and tenth passes, the microstructure is much finer and more uniform. This evolution is consistent with progressive dislocation rearrangement, subgrain formation, and conversion of deformation-induced substructures into finer grain regions. The dispersed TiB_2_ particles can interact with deformation-induced dislocations and locally constrain matrix deformation; however, given the relatively coarse starting particle size, their direct role in pinning the submicrometre grain boundaries formed after severe deformation is not assumed. Meanwhile, the increasingly continuous SAED rings are indicative of the presence of a large number of fine grains with different orientations. The indexed reflections are related to the principal crystallographic planes of the α-Al phase. HRTEM shows lattice fringes, which further indicate the coexistence of aluminium matrix and TiB_2_ reinforcement. The combined TEM results thus relate repeated ECAP processing to grain subdivision, structural homogenization, and deformation-induced substructure evolution, all supporting the observed improvement in mechanical and wear performance [62].

### Tribological behavior

Fig 12(a–e) shows the variation in wear-height loss of the AA8011−5 wt.% TiB_2_ composite with sliding distance under normal loads of 10, 20, 30, 40, and 50 N for the as-cast condition and after successive ECAP passes. In every testing condition, wear height loss increases with increasing sliding distance, while higher normal loads produce greater material removal because of the increased contact pressure at the sliding interface. However, a progressive reduction in wear height loss is evident as the number of ECAP passes increases.

**Fig 12:**
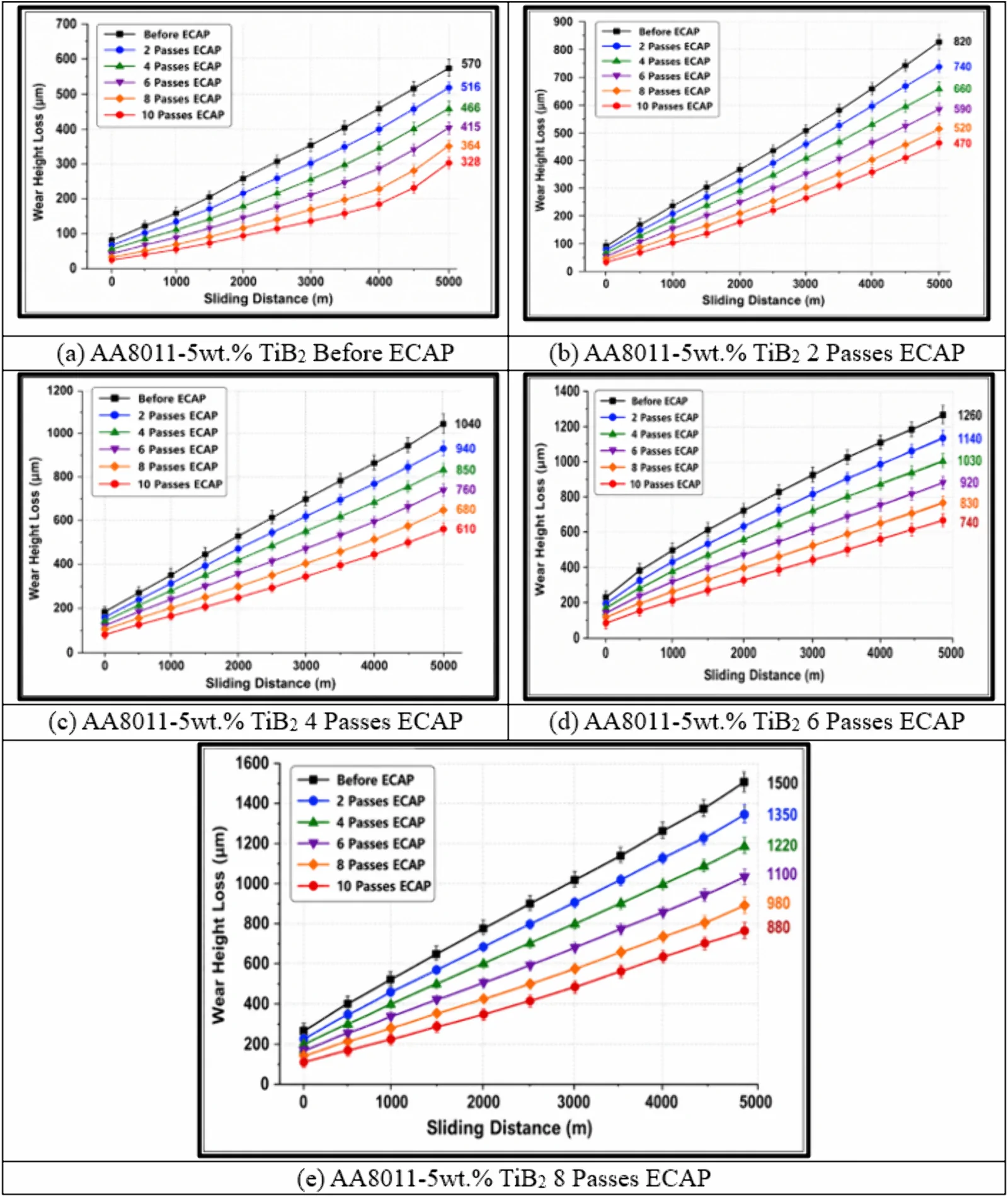
Wear-height loss of the AA8011–5 wt.% TiB_2_ composite as a function of sliding distance under normal loads of 10–50 N in the as-cast condition and after successive ECAP passes.

At a sliding distance of 5000 m under a 10 N load, the wear height loss decreases from 570 μm in the as-cast specimen to 328 μm after 10 ECAP passes. Similar decreases in wear-height loss were found with the increase of applied load. The measured values were reduced from 820 μm to 380 μm at 20 N, from 1040 μm to 610 μm at 30 N, from 1260 μm to 740 μm at 40 N, and from 1500 μm to 880 μm at 50 N. This consistent behavior indicates that the wear-related advantages of ECAP were still effective under more severe loading conditions. The increased resistance to material removal can be explained by the progressive refinement of the aluminium grains, which limited the localized plastic flow beneath the sliding surface. Simultaneously, the load support was improved and penetration of the counterface into the composite was reduced due to a more uniform distribution of TiB_2_ particles. The reduction in casting-related porosity and the improved structural continuity of the matrix–particle regions may decrease the probability of particle debonding and surface damage during sliding. Hence, the worn surface leads to reduced groove formation and material removal, and thus a stabler wear response during the test. The specimen processed for 10 ECAP passes showed the lowest wear height loss under all the applied loads among all the processing conditions. These results demonstrate that repeated ECAP substantially improves the tribological performance of the AA8011−5 wt.% TiB_2_ composite within the investigated conditions. The improvement is consistent with the combined effects of microstructural refinement, progressive densification, and more homogeneous dispersion of the reinforcement [63,64].

### Wear-rate evolution

Fig 13(a–e) shows the wear rate of the AA8011−5 wt.% TiB_2_ composite tested under normal loads of 10, 20, 30, 40 and 50 N before ECAP and after successive ECAP passes. The specific wear rate was calculated from the measured wear volume using k = ΔV/(F L), where k is the specific wear rate, ΔV is the wear volume, F is the applied normal load, and L is the sliding distance. For all loading conditions, the wear rate is initially high and then decreases gradually until it becomes almost constant as the sliding distance increases. This behaviour indicates that a stable contact layer is slowly building up on the worn surface. As the applied load increases, the wear rate increases due to the increase in contact stress. However, the wear rate of the specimens processed by ECAP is always lower than that of the as-cast composite. At a sliding distance of 5000 m, the wear rate under 10 N decreases from 0.98 × 10 ^−^ ⁶ mm^3^ N ^−^ ^1^ m ^−^ ^1^ in the as-cast specimen to 0.89, 0.76, 0.64, 0.53, and 0.44 × 10 ^−^ ⁶ mm^3^ N ^−^ ^1^ m ^−^ ¹ after 2, 4, 6, 8, and 10 ECAP passes, respectively.

**Fig 13:**
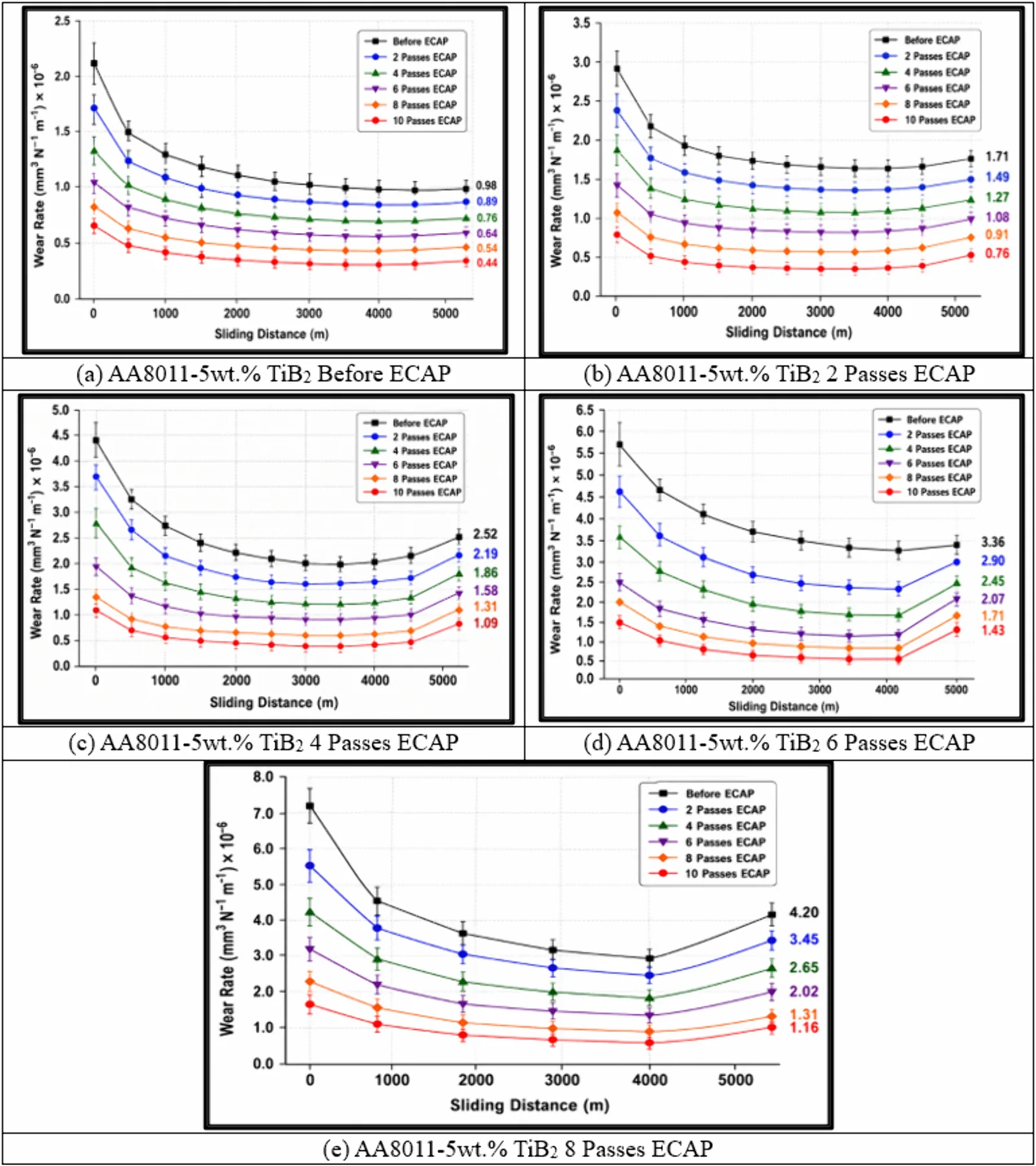
Wear rate of the AA8011–5 wt.% TiB_2_ composite as a function of sliding distance under normal loads of 10–50 N in the as-cast condition and after successive ECAP passes.

Similar improvements are obtained under 20 N, where the wear rate decreases from 1.71 to 0.76 × 10 ^−^ ⁶ mm^3^ N ^−^ ¹ m ^−^ ¹, under 30 N from 2.52 to 1.09 × 10 ^−^ ⁶ mm^3^ N ^−^ ¹ m ^−^ ¹, under 40 N from 3.36 to 1.43 × 10 ^−^ ⁶ mm^3^ N ^−^ ¹ m ^−^ ¹, and under 50 N from 4.20 to 1.76 × 10 ^−^ ⁶ mm^3^ N ^−^ ¹ m ^−^ ¹ after 10 ECAP passes. The wear rate reductions achieved are 55.1%, 55.6%, 56.7%, 57.4%, and 58.1% in comparison with the as-cast condition. The microstructural changes due to the repeated ECAP processing are closely related to the wear performance improvement. The successive deformation cycles lead to a finer grain structure, which increases the resistance of the aluminium matrix to deformation under sliding conditions [65]. The TiB_2_ particles also became more homogeneously distributed with repeated ECAP, while the improved structural integrity of the composite can contribute to more effective load sharing during sliding and reduced localized surface damage. The reduction in calculated porosity, together with the observed redistribution of TiB_2_ after ECAP, is consistent with improved structural consolidation and a reduced tendency for reinforcement pull-out during sliding. These microstructural changes may also delay the initiation and development of wear-related surface damage, thereby limiting material loss under the investigated loads. Among the conditions investigated, the composite processed with 10 ECAP passes exhibited the lowest wear rate across the applied load range. Overall, repeated ECAP was associated with improved dry-sliding performance of the AA8011–5 wt.% TiB_2_ composite within the investigated tribological conditions. However, the present observations do not independently quantify the contribution of individual mechanisms such as reinforcement pull-out, crack initiation, or load sharing to the measured wear response.

### Coefficient of friction

The coefficient of friction of the AA8011–5 wt.% TiB_2_ composite under normal loads of 10, 20, 30, 40, and 50 N in the as-cast condition and after successive ECAP passes is shown in Fig 14(a–e). At the beginning of sliding, the coefficient of friction increases sharply and then approaches a relatively steady level as the sliding distance increases, irrespective of the applied load. The initial increase is associated with the running-in stage, during which surface asperities undergo deformation and the effective contact area develops. The subsequent stabilization indicates the establishment of a relatively stable sliding interface between the composite pin and counterface.

**Fig 14:**
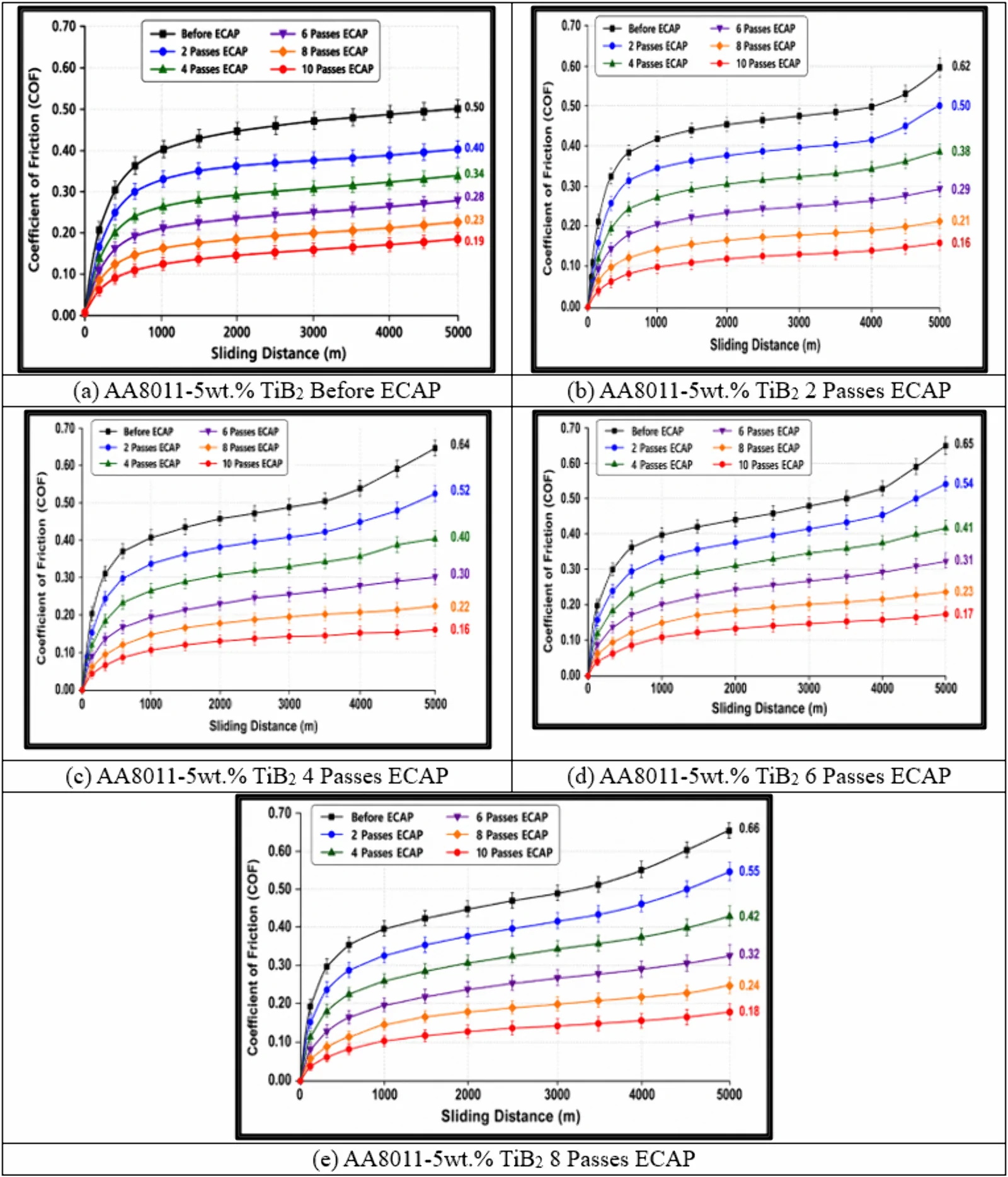
Coefficient of friction of the AA8011–5 wt.% TiB_2_ composite during 5000 m dry-sliding tests under normal loads of 10–50 N in the as-cast condition and after successive ECAP passes.

Although increasing the applied load produced higher friction coefficients, the COF decreased progressively with increasing ECAP passes. At a sliding distance of 5000 m and a normal load of 10 N, the coefficient of friction decreased from 0.50 in the as-cast condition to 0.40, 0.31, 0.24, 0.18, and 0.19 after 2, 4, 6, 8, and 10 ECAP passes, respectively. Similar reductions were observed at the higher normal loads. At 20, 30, 40, and 50 N, the COF decreased from 0.62, 0.64, 0.65, and 0.66 in the as-cast condition to 0.16, 0.16, 0.17, and 0.18, respectively, after 10 ECAP passes. Thus, repeated ECAP was associated with a progressive reduction in COF across all investigated loads, with the lowest measured value of 0.16 obtained after 10 ECAP passes at 20 and 30 N. Because the tests were performed under dry-sliding conditions without external lubrication, these values should be interpreted specifically within the present material, counterface, surface condition, normal-load, and sliding-distance conditions and should not be generalized to unlubricated aluminium contacts in general. The reduction in COF is consistent with the accompanying decrease in wear rate and the morphological changes observed on the worn surfaces. The refined grain structure can increase resistance to localized surface deformation, while the redistribution of TiB_2_ and reduction in casting-related structural discontinuities may contribute to improved surface integrity during sliding. These microstructural changes provide plausible explanations for the observed reduction in friction; however, the present experiments do not independently quantify the contribution of each mechanism. Therefore, the lower COF observed after repeated ECAP should be considered a measured response of the processed composite under the specified tribological conditions rather than direct evidence of a single dominant wear mechanism. Within the investigated processing window, the 10-pass condition exhibited the lowest COF at all applied loads, consistent with the concurrent changes in grain structure, densification, and TiB_2_ distribution [66,67].

### Worn-surface morphology

Fig 15(a–f) shows the SEM images of the worn surfaces of the AA8011–5 wt.% TiB_2_ composite after dry-sliding wear testing in the as-cast condition and after 2, 4, 6, 8, and 10 ECAP passes. The as-cast specimen shows pronounced surface degradation in the form of deep ploughing grooves, pronounced plastic deformation, surface delamination, fine wear debris, and cracked TiB_2_ particles, indicating severe wear under the applied sliding conditions. The damage is mainly associated with the relatively coarse cast microstructure and casting-induced defects, which can promote crack initiation and material removal during sliding. After 2 ECAP passes, the wear scar shows substantially lower damage severity than the as-cast sample. The worn surface exhibits finer wear grooves, less delamination, and less loose wear debris, indicating reduced material removal during sliding. This improvement is associated with the microstructural refinement produced by ECAP, together with the more homogeneous distribution of TiB_2_ particles and the improved structural continuity observed in the deformed composite. Consequently, the composite exhibits reduced surface damage and improved resistance to sliding wear. Although abrasive grooves remain visible after 2 ECAP passes, their reduced depth and extent indicate improved load-bearing capability and reduced material removal compared with the as-cast condition. After 4 ECAP passes, the worn surface shows shallower grooves together with a more uniform distribution of adhered particles and oxide patches, suggesting reduced direct interaction between the aluminium matrix and the steel counterface. After 6 ECAP passes, the worn surface exhibits fine, parallel grooves and a relatively compact surface layer with limited debris accumulation, indicating reduced surface plastic deformation during sliding. Specimens processed by 8 and 10 ECAP passes show smoother and more continuous wear tracks with very fine striations, while large delaminated regions and particle fracture are rarely observed. The development of a compact tribological surface layer provides a more stable sliding interface and may limit further subsurface damage during wear. This progressive evolution of worn-surface morphology is consistent with the refined grain structure, more homogeneous TiB_2_ dispersion, and reduction of pronounced casting-related structural discontinuities developed during repeated ECAP processing. These microstructural changes are consistent with reduced surface deformation, less severe ploughing and delamination, and a lower tendency for subsurface damage under the investigated sliding conditions. The worn-surface observations are qualitative, and quantitative parameters such as groove depth, debris-layer thickness, and the extent of particle pull-out were not independently measured. Accordingly, the specimen processed by 10 ECAP passes exhibits the highest resistance to surface degradation, consistent with the experimentally observed reductions in wear-height loss, wear rate, and coefficient of friction [68].

**Fig 15:**
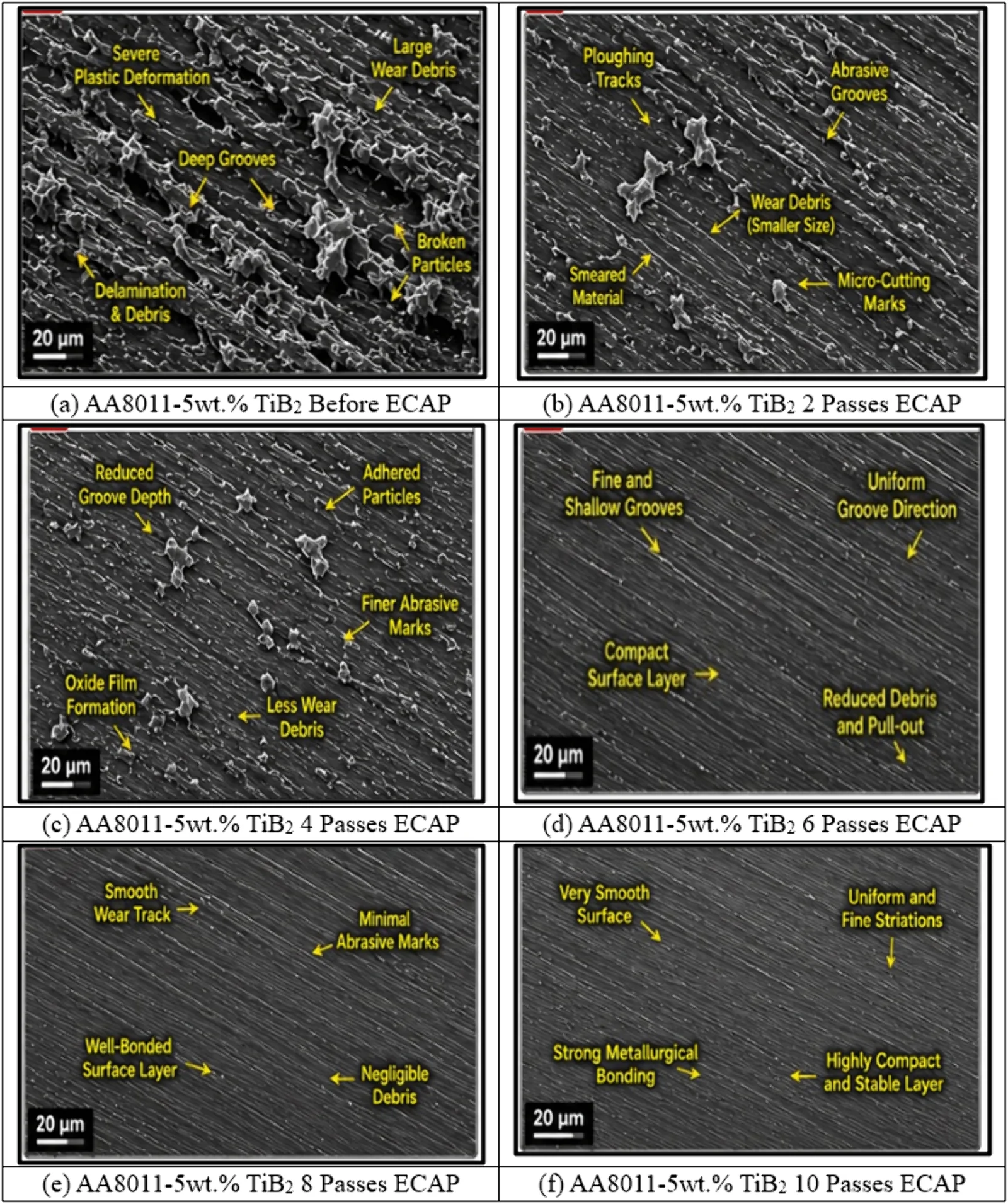
SEM images of worn surfaces of the AA8011–5 wt.% TiB_2_ composite after dry-sliding wear testing in the as-cast condition and after successive ECAP passes.

### Processing-map analysis

Fig 16(a–f) presents the processing maps of the AA8011–5 wt.% TiB_2_ composite in the as-cast condition and after 2, 4, 6, 8, and 10 ECAP passes, showing the relationship among deformation temperature, strain rate, and power-dissipation efficiency (η). The maps show a progressive change in deformation behaviour with increasing ECAP passes. In the as-cast condition, the region of high power dissipation is relatively small, while a substantial portion of the map is occupied by flow-instability regions, indicating limited deformation stability. With increasing ECAP passes, the stable deformation region progressively expands, accompanied by a reduction in the flow-instability domain.

**Fig 16:**
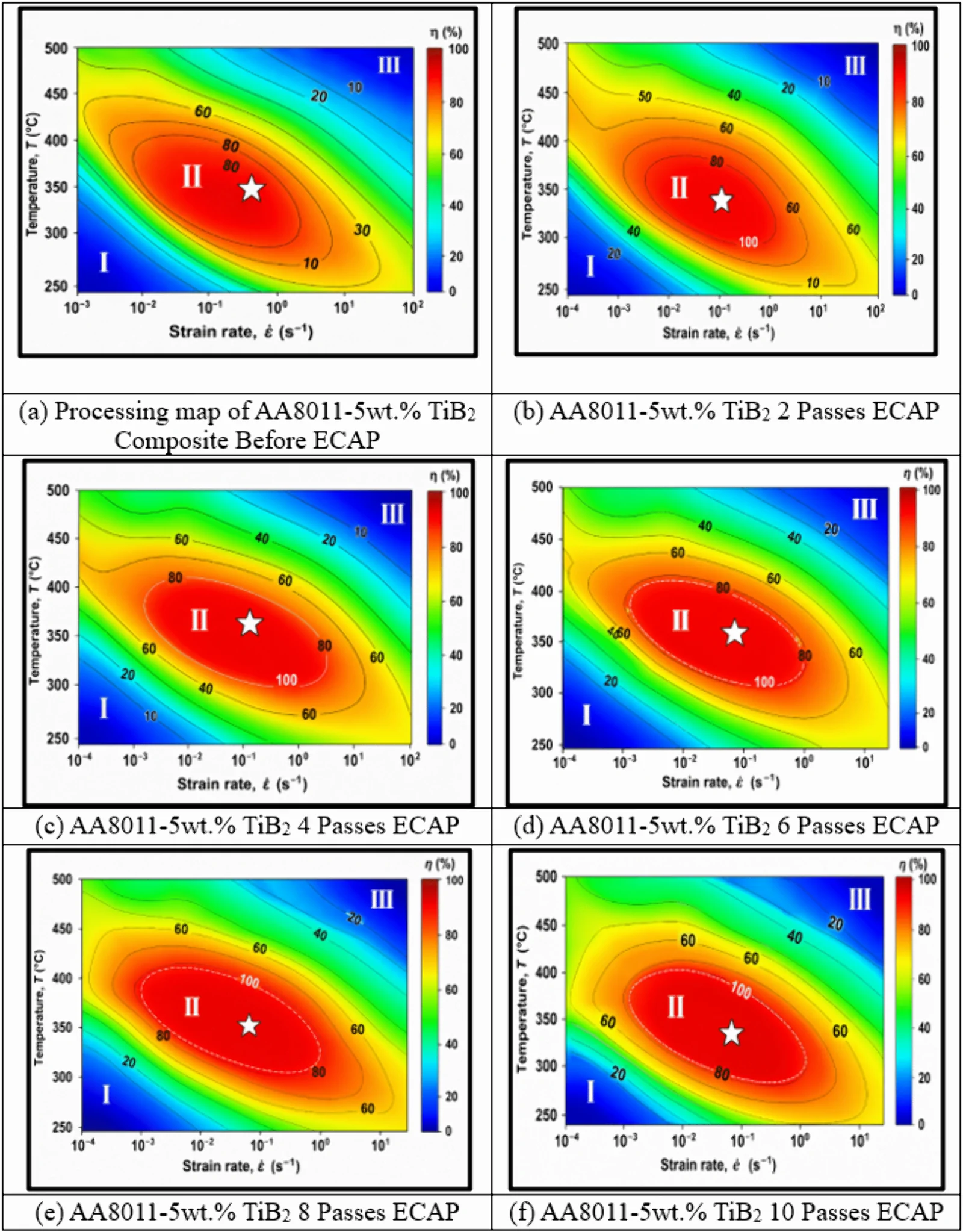
Processing maps of the AA8011–5 wt.% TiB_2_ composite showing power-dissipation efficiency and flow-instability regions in the as-cast condition and after successive ECAP passes.

The maximum power-dissipation efficiency increases systematically from approximately 80% in the as-cast condition to 86%, 90%, 93%, 96%, and 98% after 2, 4, 6, 8, and 10 ECAP passes, respectively. The calculated increase indicates a change in the DMM-derived power-dissipation response within the investigated conditions; however, the magnitude of η depends directly on the experimentally determined strain-rate sensitivity and should therefore be interpreted together with the underlying flow-stress data. A favourable stable deformation region is consistently observed approximately within 300–360 °C and 10 ^−^ ^3^–10 ^−^ ² s ^−^ ¹, where relatively stable plastic flow and high power-dissipation efficiency are indicated. The expansion of this region is consistent with improved hot-deformation stability associated with progressive microstructural refinement and homogenization. Repeated shear deformation produces a finer and more uniform grain structure, which can promote more homogeneous plastic flow during subsequent hot deformation. The TiB_2_ reinforcement can contribute to local deformation constraint and dislocation–particle interactions during thermal deformation, although the processing-map results alone do not establish a direct grain-boundary-pinning effect. The reduction of pronounced casting-related structural discontinuities and the more homogeneous TiB_2_ distribution may further reduce local strain concentrations and promote more homogeneous deformation. These observations are consistent with deformation mechanisms involving dynamic recovery and, potentially, dynamic recrystallization; however, the processing maps alone do not establish the occurrence of a specific recrystallization mechanism. The specimen processed through 10 ECAP passes exhibited the widest stable deformation region and the highest calculated power-dissipation efficiency among the investigated conditions, indicating improved hot-deformation stability within the evaluated temperature and strain-rate ranges. Overall, the processing maps indicate that repeated ECAP is associated with changes in the calculated deformation-stability field within the investigated temperature and strain-rate ranges. The maps therefore provide a comparative assessment of the hot-deformation response of the different ECAP conditions rather than a direct validation of industrial hot-forming performance. In particular, the identified high-efficiency regions should be interpreted together with the underlying flow-stress data and the assumptions associated with numerical differentiation. Dedicated hot-forming experiments are required to confirm the applicability of these processing windows under component-relevant conditions [69,70].

### Integrated interpretation of the processing–microstructure–property relationship

The experimental results provide mutually consistent evidence for the influence of accumulated ECAP deformation on the AA8011−5 wt.% TiB_2_ composite. EBSD quantitatively records a reduction in mean grain size from 12.45 to 0.54 μm, while the TEM observations independently show the transition from coarse grains and deformation-induced substructures to a much finer equiaxed structure. The optical and SEM observations further indicate progressive redistribution of TiB_2_ particles and reduction of pronounced particle-rich regions. These microstructural changes occur concurrently with systematic increases in hardness, tensile strength, compressive strength, flexural strength, impact energy, and elongation. The wear measurements provide an additional functional validation, with both wear rate and coefficient of friction decreasing progressively with increasing ECAP passes. The processing maps further show a concurrent expansion of the stable deformation region. The agreement among independent microstructural, mechanical, tribological, and hot-deformation measurements supports the proposed processing–microstructure–property relationship, while the individual strengthening and wear mechanisms are interpreted as contributing mechanisms rather than quantitatively isolated effects.

### Scope and limitations

The present investigation was intentionally focused on a fixed TiB_2_ content of 5 wt.%, a single ECAP die geometry, route BC, and ECAP pass numbers up to 10. Consequently, the 10-pass condition represents the best-performing condition within the investigated processing window and should not be interpreted as a global optimum. The effects of reinforcement content, alternative ECAP routes, different die geometries, and pass numbers beyond 10 were not established.

An unreinforced AA8011 control processed under identical casting and ECAP conditions was not included. Therefore, the present study establishes the evolution of the AA8011–5 wt.% TiB_2_ composite with increasing ECAP deformation but does not independently separate the contribution of TiB_2_ from the effect of ECAP relative to an unreinforced reference.

The tribological assessment was conducted under the specified dry-sliding conditions, normal loads of 10–50 N, and a maximum sliding distance of 5000 m. The observed wear response therefore represents the investigated operating window rather than all possible contact conditions. Worn-surface SEM observations provide morphological support for the measured wear behaviour, but individual contributions from abrasive wear, delamination, oxidation, and reinforcement pull-out were not independently quantified.

Similarly, the hot-deformation response was evaluated using Dynamic Materials Model-based processing maps derived from compression data. The calculated power-dissipation efficiency and stability fields should therefore be interpreted within the experimentally measured temperature and strain-rate domain and together with the underlying flow-stress data and numerical differentiation procedure. Dedicated hot-forming experiments are required to validate the identified processing windows under component-relevant conditions.

The study also does not independently quantify the contributions of Hall–Petch strengthening, dislocation strengthening, particle strengthening, and load transfer. These mechanisms are therefore discussed as concurrent contributors based on the measured microstructural and mechanical trends. Corrosion, fatigue, thermal cycling, and long-duration service performance were not directly evaluated. Future studies should examine reinforcement-content effects, unreinforced AA8011 controls, alternative ECAP routes and pass schedules, quantitative particle-distribution metrics, independent strengthening contributions, and component-scale durability.

## Conclusions

The present study investigated the effect of multi-pass ECAP on the microstructure, mechanical performance, tribological behaviour, and hot-deformation response of AA8011−5 wt.% TiB_2_ composites produced by ultrasonic-assisted stir casting. The results demonstrate that the combined processing route progressively refined the microstructure, improved TiB_2_ distribution and structural consolidation, and produced corresponding improvements in mechanical and dry-sliding performance. The principal conclusions obtained from the experimental investigation are summarized below.

- Ultrasonic-assisted stir casting followed by multi-pass Equal Channel Angular Pressing (ECAP) was successfully employed to produce AA8011−5 wt.% TiB_2_ composites. The combined processing route promoted particle incorporation and subsequent microstructural refinement, providing a basis for improving the mechanical, tribological, and hot-deformation response of the composite.
- ECAP produced progressive grain refinement and improved TiB_2_ distribution. After 10 ECAP passes, the ultimate tensile strength increased from 182 to 266 MPa, corresponding to an increase of 46.2% relative to the as-cast condition. Hardness, compressive strength, flexural strength, impact energy, and elongation also increased progressively with ECAP passes. The observed improvements are consistent with the combined effects of grain-boundary strengthening, deformation-induced dislocation strengthening, improved load transfer, reduced the influence of casting-related structural discontinuities, and particle–matrix constraint and dislocation–particle interactions. These mechanisms act concurrently, and their individual contributions cannot be quantitatively separated from the present experimental data.
- ECAP substantially improved the tribological response within the investigated dry-sliding conditions. After 10 ECAP passes, the wear rate decreased by approximately 55–58% and the coefficient of friction decreased by approximately 62–75% relative to the as-cast condition across the applied load range of 10–50 N at a sliding distance of 5000 m. The improvement is consistent with the combined effects of grain refinement, progressive densification, more homogeneous TiB_2_ distribution, and improved resistance to surface damage.
- XRD, optical microscopy, SEM, EDS, EBSD, and TEM collectively demonstrated progressive structural refinement during ECAP. In particular, the EBSD-measured mean grain size decreased from 12.45 μm in the as-cast condition to 0.54 μm after 10 ECAP passes, while the microstructural observations indicated progressive redistribution of TiB_2_ and reduction of pronounced particle-rich regions. These changes provide a consistent microstructural basis for the measured mechanical and tribological improvements.
- The combined ultrasonic-assisted stir-casting and multi-pass ECAP route was effective in tailoring the structural and functional response of the AA8011−5 wt.% TiB_2_ composite. Ultrasonic-assisted casting established the initial composite structure, whereas subsequent ECAP promoted grain refinement, increased material consolidation, and reinforcement redistribution. Within the investigated processing conditions, the combined route therefore provides a promising approach for developing lightweight aluminium matrix composites with improved mechanical and dry-sliding performance.
- Processing-map analysis indicated a progressive expansion of the stable hot-deformation region with increasing ECAP passes. The maximum power-dissipation efficiency increased from approximately 80% in the as-cast condition to 98% after 10 ECAP passes, corresponding to an increase of approximately 22.5%. The reduced instability regions and expanded stable domain indicate improved hot-deformation stability within the investigated temperature and strain-rate ranges. The processing-map results therefore support the potential of multi-pass ECAP for improving the hot-workability of AA8011−5 wt.% TiB_2_ under the investigated conditions, while the specific underlying deformation mechanisms require further experimental verification.

## Supporting information

S1 File Supporting Information. Processed numerical data underlying the quantitative results reported in the manuscript, including mechanical properties, EBSD grain-size calculations, density and porosity, coefficient of friction, wear rate, processing-map data, and wear-height loss.
(DOCX)

## References

[1] Agarwal A, Mthembu L. Weight optimization of heavy-duty truck chassis by optimal space fill design using light weight graphite Al GA 7-230 MMC. Mater Today Proc. 2021. doi: 10.1016/j.matpr.2021.11.053

[2] Agarwal A, Mthembu L. Structural analysis and weight optimization of automotive chassis by Latin hypercube sampling using metal matrix composites. Mater Today Proc. 2022;60:2132–40. doi: 10.1016/j.matpr.2022.02.059

[3] Makki E, Thakur S, Samal P, Kumar A, Singh J, Kumar R, et al. Investigation of microstructure, mechanical properties and fracture behaviour of AA8011/TiB2 composites for sustainable engineering applications. Sci Rep. 2026;16(1):24679. doi: 10.1038/s41598-026-55567-w 42215671 PMC13454612

[4] Ilunga M, Agarwal A. A finite-element-analysis-based feasibility study for optimizing pantograph performance using aluminum metal matrix composites. Processes. 2024;12(3):445. doi: 10.3390/pr12030445

[5] Agarwal A, Molwane OB, Marumo R. Design optimization of knuckle stub using response surface optimization. Springer Proceedings in Materials. Springer Singapore. 2020. p. 155–64. doi: 10.1007/978-981-15-7827-4_15

[6] Prince M. Microstructural and mechanical evaluation of stir-cast AA8011 composites with 2% TiB2 and variable SiC. J Ceramic Process Res. 2026;27. doi: 10.36410/jcpr.2026.27.3.406

[7] Medveď D, Andrejovská J, Puchý V, Džunda R, Vojtko M. Tribological response of TiC- and (W,Ti)C-reinforced Al_2_O_3_ ZrO_2_ composites: Role of reinforcement homogeneity and tribolayer stability. J Eur Ceram Soc. 2027;47:118807. doi: 10.1016/j.jeurceramsoc.2026.118807

[8] Sankarasabapathi S, Subramaniam N, Velmurugan S, Nelson GRE. Exploring the mechanical and microstructural characterization of stir cast aluminum 8011 matrix hybrid composites. Matéria (Rio J). 2025;30. doi: 10.1590/1517-7076-rmat-2024-0904

[9] Munivenkatappan MSB, Shanmugam S, Veeramani A. Synthesis and aharacterization of In-Situ AA8011-TiB2 composites produced by flux assisted synthesis. ACSM. 2020;44(5):333–8. doi: 10.18280/acsm.440505

[10] Abishkenov M, Tavshanov I, Lutchenko N, Nogayev K, Ashkeyev Z, Kulidan S. Microstructure and mechanical properties of Al6060/TiB2 Aluminum matrix composites produced via ultrasonically assisted stir casting and radial-shear rolling. JMMP. 2025;9(9):309. doi: 10.3390/jmmp9090309

[11] Gowthami G, Vemula J. Fabrication and wear analysis of Al 8009-TiB2 composites produced by ultrasonic assisted stir casting. Int J Interact Des Manuf. 2024;19(8):5601–20. doi: 10.1007/s12008-024-02152-y

[12] Xie Z, Jiang R, Li X, Zhang L, Li A, He Z. Microstructural evolution and mechanical properties of TiB2/2195 composites fabricated by ultrasonic-assisted in-situ casting. Ultrason Sonochem. 2022;90:106203. doi: 10.1016/j.ultsonch.2022.106203 36265292 PMC9579803

[13] Zhang N, Zhang J, Jiang F, Li L, Wang X, Yuan Z, et al. Continuous multi-scale structural analysis and strengthening mechanisms of spray deposited TiB2/Al-Cu composites. J Alloys Compd. 2026;1051:185939. doi: 10.1016/j.jallcom.2026.185939

[14] Pole M, Mukhopadhyay S, Kastamo S, Loukus A, Choi JP, Olszta M, et al. Tribological behavior of hybrid Aluminum-TiB2 metal matrix composites for brake rotor applications. Wear. 2025;562–563:205639. doi: 10.1016/j.wear.2024.205639

[15] Farooq SA, Mukhtar SH, Raina A, Ul Haq MI, Siddiqui MIH, Naveed N, et al. Effect of TiB2 on the mechanical and tribological properties of marine grade Aluminum Alloy 5052: An experimental investigation. J Mater Res Technol. 2024;29:3749–58. doi: 10.1016/j.jmrt.2024.02.106

[16] Pang B, He Z, Liu Z, Zhao X, Lu K, Zhao Y, et al. Thermal deformation behavior and microstructural evolution of AlZnMgCu/TiB_2_ alloy. J Alloys Compd. 2025;1042:184138. doi: 10.1016/j.jallcom.2025.184138

[17] Deng H, Zheng Z, Song W, Tan X, Liang X, Li H, et al. Constitutive model and microstructure evolution of thermal deformation behavior of in situ TiB_2_p/Al-Zn-Mg-Cu composites with high Zn content. Mater Today Commun. 2024;41:110308. doi: 10.1016/j.mtcomm.2024.110308

[18] Huang J, Xiang Z, Li M, Li L, Chen Z. Hot deformation behavior and microstructural evolution of a TiB2/Al-Zn-Mg-Cu-Zr composite. Materials. 2024;17:1487. doi: 10.3390/ma17071487 PMC11012281 38612001

[19] Sharath B, Madhu P, Verma A. Wear behaviour of aluminium-based hybrid composites processed by equal channel angular pressing. Proceedings of the Institution of Mechanical Engineers, Part J: Journal of Engineering Tribology. 2024;238(9):1079–90. doi: 10.1177/13506501241247616

[20] Awasthi A, Gupta A, Saxena KK, Diwedi RK. Equal channel angular processing on aluminium and its alloys – A review. Mater Today Proc. 2022;56:2388–91. doi: 10.1016/j.matpr.2021.12.207

[21] Andilab B, Emadi P, Roy R, Ravindran C. Ultrasonic processing of lightweight alloys: A critical review. Int Mater Rev. 2025;71(1):3–29. doi: 10.1177/09506608251369176

[22] Ramani S, Wins KLD, Nampoothiri J, Ravi KR, Dhas DSEJ. Effect of post-reaction ultrasonic treatment on synthesis, microstructural evolution and mechanical behaviour of Al 4043/TiB2 in situ nanocomposites. Arab J Sci Eng. 2021;46:7521–31. doi: 10.1007/s13369-021-05468-z

[23] Sheelwant A, Jadhav PM, Narala SKR. ANN-GA based parametric optimization of Al-TiB2 metal matrix composite material processing technique. Mater Today Commun. 2021;27:102444. doi: 10.1016/j.mtcomm.2021.102444

[24] Sun W, Xiang Z, Li J, Yang Z, Han Y, Chen Z. Effect of TiB2 content on microstructure and mechanical properties of TiB2/Al-Zn-Mg-Cu composites with High Zn content. Materials (Basel). 2025;18(22):5191. doi: 10.3390/ma18225191 41304035 PMC12654510

[25] Li X, Zhao K, Yang L, Guo E, Kang H, Zhang Y, et al. Synergetic effects of trace Sc/Zr/TiB 2 on recrystallization and strengthening behavior of Al–Mg alloys. Rare Metals. 2025;44(5):3514–30. doi: 10.1007/s12598-024-03080-2

[26] Jia C, Xu Z, Li Y, He Y, Liu H, Zhu Z. Hot deformation behavior and microstructural evolution of an in situ 2 wt.% TiB2-reinforced 6061 Al matrix composite. J Mater Eng Perform. 2024;33:3309–19. doi: 10.1007/s11665-023-08226-4

[27] Xie PP, Liu ZW, Chen M, Yu SW, He Y. Hot deformation behavior and hot processing map of Sc doped TiB2/6061 composite. Zhongguo Youse Jinshu Xuebao/Chinese Journal of Nonferrous Metals. 2023;33. doi: 10.11817/j.ysxb.1004.0609.2022-43119

[28] Pai BC, Ramani G, Pillai RM, Satyanarayana KG. Role of magnesium in cast aluminium alloy matrix composites. J Mater Sci. 1995;30:1903–11. doi: 10.1007/BF00353012

[29] Meti VKV, Shirur S, Nampoothiri J, Ravi KR, Siddhalingeshwar IG. Synthesis, characterization and mechanical properties of AA7075 based MMCs reinforced with TiB2 particles processed through ultrasound assisted in-situ casting technique. Trans Indian Inst Met. 2017;71(4):841–8. doi: 10.1007/s12666-017-1216-5

[30] Huang K, Jiang R, Li X, Zhang L, Li Z, Li R. Effects of high-intensity ultrasound on microstructure and mechanical property of in situ TiB2/2A14 composites. Metals. 2019;9(11):1210. doi: 10.3390/met9111210

[31] Maddaiah KC, Kumar GBV, Pramod R. Studies on the mechanical, strengthening mechanisms and tribological characteristics of AA7150-Al_2_O_3_ nano-metal matrix composites. J Compos Sci. 2024;8(3):97. doi: 10.3390/jcs8030097

[32] Chidambaram A, Balasivanandha Prabu S, Padmanabhan KA. Microstructure and mechanical properties of AA6061–5wt. %TiB2 in-situ metal matrix composite subjected to equal channel angular pressing. Mater Sci Eng. 2019;759:762–9. doi: 10.1016/j.msea.2019.05.068

[33] ASTM International. ASTM E8/E8M-25: Standard Test Methods for Tension Testing of Metallic Materials. doi: 10.1520/E0008_E0008M-25

[34] ASTM International. ASTM E9-19: Standard Test Methods of Compression Testing of Metallic Materials at Room Temperature. West Conshohocken, PA: ASTM International. 2019. doi: 10.1520/E0009-19

[35] ASTM International. ASTM E23-25: Standard Test Methods for Notched Bar Impact Testing of Metallic Materials. West Conshohocken, PA. 2025. doi: 10.1520/E0023-25

[36] ASTM International. ASTM G99-23: Standard Test Method for Wear and Friction Testing with a Pin-on-Disk or Ball-on-Disk Apparatus. West Conshohocken, PA: ASTM International. 2023. doi: 10.1520/G0099-23

[37] ASTM International. ASTM E209-18: Standard Practice for Compression Tests of Metallic Materials at Elevated Temperatures with Conventional or Rapid Heating Rates and Strain Rates. West Conshohocken, PA. 2018. doi: 10.1520/E0209-18

[38] M S, B R, S RK. Low cycle fatigue behaviour of aluminium 6063 alloy processed by equal channel angular pressing at 90° and 120° channel angles. Mater Today Commun. 2025;42:111401. doi: 10.1016/j.mtcomm.2024.111401

[39] Awasthi A, Sathish Rao U, Saxena KK, Dwivedi RK. Impact of equal channel angular pressing on aluminium alloys: An overview. Mater Today Proc. 2022;57:908–12. doi: 10.1016/j.matpr.2022.03.037

[40] Tang J, Yi Y, He H, Huang S, Zhang J, Dong F. Hot deformation behavior and microstructural evolution of the Al-Cu-Li alloy: A study with processing map. JJ Alloys Compd. 2023;934:167755. doi: 10.1016/j.jallcom.2022.167755

[41] Xu W, Jin X, Xiong W, Zeng X, Shan D. Study on hot deformation behavior and workability of squeeze-cast 20 vol%SiCw/6061Al composites using processing map. Mater Charact. 2018;135:154–66. doi: 10.1016/j.matchar.2017.11.026

[42] Guan R, Misra RDK, Shang Y, An Y, Wang Y, Zhang Y, et al. Mechanism of microstructural refinement of deformed aluminum under synergistic effect of TiAl3 and TiB2 particles and impact on mechanical properties. Mater Sci Eng. 2018;716:129–39. doi: 10.1016/j.msea.2018.01.043

[43] Mallikarjuna C, Shashidhara SM, Mallik US, Parashivamurthy KI. Grain refinement and wear properties evaluation of aluminum alloy 2014 matrix-TiB2 in-situ composites. Mater Des. 2011;32:3554–9. doi: 10.1016/j.matdes.2011.01.036

[44] Tian C, Chen L, Chen Y, Li Z, Qian L, Zhao G. Effects of multi-directional forging on the microstructure and mechanical properties of TiB_2_ particulate reinforced 6061Al composite. J Mater Res Technol. 2023;27:1945–57. doi: 10.1016/j.jmrt.2023.10.069

[45] Zhao S, Zhang H, Cui Z, Chen D, Chen Z. Particle dispersion and grain refinement of in-situ TiB_2_ particle reinforced 7075 Al composite processed by elliptical cross-section torsion extrusion. J Alloys Compd. 2020;834:155136. doi: 10.1016/j.jallcom.2020.155136

[46] Korchef A, Souid I. Grain refinement and strengthening of an aluminum alloy subjected to severe plastic deformation through equal-channel angular pressing. Crystals. 2023;13(8):1160. doi: 10.3390/cryst13081160

[47] Snopiński P, Appiah ANS, Hilšer O, Kotoul M. Investigation of microstructure and mechanical properties of SLM-fabricated AlSi10Mg alloy post-processed using Equal Channel Angular Pressing (ECAP). Materials (Basel). 2022;15(22):7940. doi: 10.3390/ma15227940 36431429 PMC9697299

[48] Injor OM, Daramola OO, Adewuyi BO, Adediran AA, Ramakokovhu MM, Sadiku RE, et al. Grain refinement of Al–Zn–Mg alloy during equal channel angular pressing (ECAP). Results in Engineering. 2022;16:100739. doi: 10.1016/j.rineng.2022.100739

[49] Bhatnagar AS, Gupta A, Arora G, Padmanabhan S, Burela RG. Mean-field homogenization coupled low-velocity impact analysis of nano fibre reinforced composites. Mater Today Commun. 2021;26:102089. doi: 10.1016/j.mtcomm.2021.102089

[50] Bhowmik P, Arora G. Graphene/Metal Matrix Nanocomposites. Metal Matrix Composites. Boca Raton: CRC Press. 2022. p. 109–48. doi: 10.1201/9781003194910-5

[51] Agarwal A, Rimal A. Structural and dynamic evaluation of an agricultural power take-off shaft using lightweight metal matrix composites. Metal Matrix Composites. Elsevier. 2026. p. 467–87. doi: 10.1016/b978-0-443-40371-2.00015-5

[52] Elibol C, Sagir K, Dogan M. Effect of equal-channel angular pressing on microstructure, aging kinetics and impact behavior in a 7075 aluminum alloy. Mater Today Commun. 2024;39:108931. doi: 10.1016/j.mtcomm.2024.108931

[53] Abd El Aal MI. Influence of equal channel angular pressing and cyclic extrusion compression on the microstructure evolution and mechanical properties of pure aluminum. Materials (Basel). 2024;17(20):5061. doi: 10.3390/ma17205061 39459766 PMC11509442

[54] Mireles-Ramos A, García-Pastor F. Crystallographic texture evolution of aluminum samples with a rectangular cross-section during equal channel angular pressing processing. Metals. 2024;14(12):1460. doi: 10.3390/met14121460

[55] Pouyafar V, Malihi F, Meshkabadi R. Conjoint influence of equal channel angular pressing and annealing on microstructure and mechanical properties of aluminum alloy 7075. Materwiss Werksttech. 2024;55(11):1590–600. doi: 10.1002/mawe.202300379

[56] Chegini M, Shaeri MH, Taghiabadi R, Chegini S, Djavanroodi F. The Correlation of microstructure and mechanical properties of in-situ Al-Mg2Si cast composite processed by equal channel angular pressing. Materials (Basel). 2019;12(9):1553. doi: 10.3390/ma12091553 31083626 PMC6540018

[57] Arab MS, El Mahallawy N, Shehata F, Agwa MA. Refining SiCp in reinforced Al–SiC composites using equal-channel angular pressing. Mater Des. 2014;64:280–6. doi: 10.1016/j.matdes.2014.07.045

[58] Tolcha MA, Gebrehiwot TM, Lemu HG. Enhancing mechanical properties of cast ingot Al6061 alloy using ECAP process. J Mater Eng Perform. 2024;33:13553–66. doi: 10.1007/s11665-024-09978-3

[59] Snopiński P, Woźniak A, Pagáč M. Microstructural evolution, hardness, and strengthening mechanisms in SLM AlSi10Mg alloy subjected to equal-channel angular pressing (ECAP). Materials. 2021;14:7598. doi: 10.3390/ma14247598 PMC8708603 34947192

[60] Snopiński P, Król M, Pagáč M, Petrů J, Hajnyš J, Mikuszewski T, et al. Effects of equal channel angular pressing and heat treatments on the microstructures and mechanical properties of selective laser melted and cast AlSi10Mg alloys. Archiv Civ Mech Eng. 2021;21(3). doi: 10.1007/s43452-021-00246-y

[61] He X, Dong F, Zhang Y. Improving the microstructure and mechanical properties of Al-0.7Fe-0.4Mg-0.1Si-0.5Er alloy by equal channel angular pressing. Materials. 2025;18:4007. doi: 10.3390/ma18174007 PMC12428854 40942440

[62] Liu Y, Xu J, Chen B, Fan Y, Lv W, Sun H. The influence of equal-channel angular pressing on the microstructure and properties of a steel–aluminum composite. Metals. 2025;15(7):774. doi: 10.3390/met15070774

[63] Gruber M, Illgen C, Lichte F, Hartmann C, Frint P, Wagner MF-X, et al. Establishing equal-channel angular pressing (ECAP) for sheet metals by using backpressure: manufacturing of high-strength aluminum AA5083 sheets. Int J Adv Manu Technol. 2023;127:3481–95. doi: 10.1007/s00170-023-11692-8

[64] Syukron M, Anasyida AS, Zuhailawati H, Hassan MH, Dhindaw BK, Zakaria SA, et al. Enhancing microstructure, grain refinement, and wear properties of cast A356-TiB2 composite through improved sequence of ECAP and heat treatment processes. J min metall B Metall. 2024;60(3):435–50. doi: 10.2298/jmmb240111035s

[65] A C, Vivekananda AS, Shanmugavel BP, Padmanabhan KA. On the wear behavior of AA6061 alloy and in-situ AA6061-TiB_2_ composite subjected to equal channel angular pressing. Surf Topogr. 2020;8:045005. doi: 10.1088/2051-672X/abbb80

[66] Alateyah AI, Alinizzi M, Zein H, Kouta H, Shaban M, Alawad MO, et al. Optimizing wear behavior of AA7075 processed via ECAP: An integrated experimental, computational, and machine learning approach. Mater Today Commun. 2025;47:113075. doi: 10.1016/j.mtcomm.2025.113075

[67] Radhakrishna Bhat BV, Mahajan YR, Roshan HM, Prasad YVRK. Processing map for hot working of 6061 Al–10 vol.-% Al_2_O_3_ metal matrix composite. Mater Sci Technol. 1995;11(2):167–73. doi: 10.1179/mst.1995.11.2.167

[68] Elhefnawey M, Shuai GL, Li Z, Nemat-Alla M, Zhang DT, Li L. On dry sliding wear of ECAPed Al-Mg-Zn alloy: Wear rate and coefficient of friction relationship. Alexandria Eng J. 2021;60(1):927–39. doi: 10.1016/j.aej.2020.10.021

[69] Fakhar N, Khademi E, Momeni A. High temperature behavior of severely deformed AA 5083 through equal channel lateral extrusion. Mater Chem Phys. 2020;243:122581. doi: 10.1016/j.matchemphys.2019.122581

[70] Arun MS, Chakkingal U. A constitutive model to describe high temperature flow behavior of AZ31B magnesium alloy processed by equal-channel angular pressing. Mater Sci Eng. 2019;754:659–73. doi: 10.1016/j.msea.2019.03.106

