# Supplementary material for: Experimental investigation of microstructural and performance evolution in ultrasonic-assisted stir-cast AA8011–5 wt.% TiB2 composites during multi-pass equal-channel angular pressing
Source: PLoS One. 2026 Oct 28;21(10):e0361373. doi: 10.1371/journal.pone.0361373 (PMC13716218; doi:10.1371/journal.pone.0361373)
Supplement: Supplementary file 1 [file pone.0361373.s001.docx]

**Supporting Information**

**Experimental Investigation of Microstructural and Performance Evolution in Ultrasonic-Assisted Stir-Cast AA8011–5 wt.% TiB₂ Composites during Multi-Pass Equal-Channel Angular Pressing**

This Supporting Information provides the processed numerical data underlying the quantitative tables and plots reported in the manuscript. The file contains the values used for mechanical properties, EBSD grain-size calculations, density/porosity, tribological measurements, and processing-map contours. It does not include unrelated laboratory records or raw instrument files.

**Data availability.** All data underlying the findings reported in this article are provided in the Supporting Information files. Values are presented in the same form and units used for the manuscript analyses.

# Table S1. Mechanical-property data

| **ECAP Passes** | **Hardness (HV) Mean ± SD** | **UTS (MPa) Mean ± SD** | **Compression (MPa) Mean ± SD** | **Impact (J) Mean ± SD** | **Flexural (MPa) Mean ± SD** | **Elongation (%) Mean ± SD** |
| --- | --- | --- | --- | --- | --- | --- |
| 0 | 74 ± 3 | 182 ± 8 | 298 ± 10 | 5.8 ± 0.25 | 248 ± 7 | 8.2 ± 0.4 |
| 2 | 82 ± 4 | 206 ± 10 | 327 ± 12 | 6.4 ± 0.3 | 274 ± 8 | 10.1 ± 0.5 |
| 4 | 89 ± 4 | 224 ± 10 | 352 ± 13 | 7.1 ± 0.3 | 298 ± 9 | 11.8 ± 0.5 |
| 6 | 95 ± 4 | 241 ± 11 | 378 ± 14 | 7.8 ± 0.35 | 321 ± 10 | 13.0 ± 0.6 |
| 8 | 101 ± 5 | 254 ± 11 | 396 ± 15 | 8.4 ± 0.35 | 338 ± 10 | 14.2 ± 0.6 |
| 10 | 107 ± 5 | 266 ± 12 | 415 ± 16 | 8.9 ± 0.4 | 352 ± 11 | 15.1 ± 0.7 |

*Source: mechanical properties.xlsx. Values are reported as mean ± SD as provided in the source workbook.*

# Table S2. EBSD grain-size and grain-count calculations

| **ECAP Passes** | **Mean Grain Size d̄ (µm)** | **Detected Grains n** | **Grain Size Reduction (%)** | **Grain Refinement Ratio (d0/dN)** | **Grain Number Increase (%)** | **Grain Number Ratio (nN/n0)** |
| --- | --- | --- | --- | --- | --- | --- |
| 0 | 12.45 | 280 | 0 | 1 | 0 | 1 |
| 2 | 6.32 | 520 | 49.23694779116465 | 1.969936708860759 | 85.71428571428571 | 1.857142857142857 |
| 4 | 3.18 | 980 | 74.4578313253012 | 3.915094339622641 | 250 | 3.5 |
| 6 | 1.72 | 1660 | 86.18473895582329 | 7.238372093023256 | 492.8571428571429 | 5.928571428571429 |
| 8 | 0.96 | 2450 | 92.28915662650601 | 12.96875 | 775 | 8.75 |
| 10 | 0.54 | 3580 | 95.66265060240966 | 23.05555555555555 | 1178.571428571429 | 12.78571428571429 |

*Source: AA8011-5TiB2-ECAP-EBSD gain distribution -Calculations.xlsx.*

# Table S3. Density and calculated porosity

| **ECAP Passes** | **Condition** | **Theoretical Density (g/cm³)** | **Experimental Density (g/cm³)** | **Density Difference (g/cm³)** | **Calculated Porosity (%)** | **Relative Density (%)** | **Porosity Reduction vs. Before ECAP (%)** |
| --- | --- | --- | --- | --- | --- | --- | --- |
| 0 | Before ECAP | 2.86 | 2.79 | 0.06999999999999984 | 2.447552447552442 | 97.55244755244756 | 0 |
| 2 | 2 Passes ECAP | 2.86 | 2.81 | 0.04999999999999982 | 1.748251748251742 | 98.25174825174827 | 28.57142857142867 |
| 4 | 4 Passes ECAP | 2.86 | 2.82 | 0.04000000000000004 | 1.3986013986014 | 98.6013986013986 | 42.85714285714267 |
| 6 | 6 Passes ECAP | 2.86 | 2.83 | 0.0299999999999998 | 1.048951048951042 | 98.95104895104896 | 57.14285714285732 |
| 8 | 8 Passes ECAP | 2.86 | 2.84 | 0.02000000000000002 | 0.6993006993007 | 99.3006993006993 | 71.42857142857133 |
| 10 | 10 Passes ECAP | 2.86 | 2.85 | 0.009999999999999787 | 0.3496503496503422 | 99.65034965034965 | 85.71428571428599 |

*Source: density and porasity.xlsx. Porosity is the calculated bulk density difference relative to the stated theoretical density.*

# Tables S4–S8. Coefficient of friction data

**Table S4. COF at 10 N: sliding-distance data (mean and error term)**

| **Sliding Distance (m)** | **Before ECAP Mean COF** | **Before ECAP Error ±** | **2 Passes ECAP Mean COF** | **2 Passes ECAP Error ±** | **4 Passes ECAP Mean COF** | **4 Passes ECAP Error ±** | **6 Passes ECAP Mean COF** | **6 Passes ECAP Error ±** | **8 Passes ECAP Mean COF** | **8 Passes ECAP Error ±** | **10 Passes ECAP Mean COF** | **10 Passes ECAP Error ±** |
| --- | --- | --- | --- | --- | --- | --- | --- | --- | --- | --- | --- | --- |
| 0 | 0.01 | 0.005 | 0.01 | 0.005 | 0.01 | 0.005 | 0.01 | 0.005 | 0.01 | 0.005 | 0.01 | 0.005 |
| 500 | 0.31 | 0.018 | 0.24 | 0.015 | 0.19 | 0.012 | 0.15 | 0.01 | 0.12 | 0.009 | 0.09 | 0.008 |
| 1000 | 0.4 | 0.015 | 0.33 | 0.013 | 0.26 | 0.011 | 0.21 | 0.01 | 0.16 | 0.008 | 0.12 | 0.007 |
| 1500 | 0.43 | 0.014 | 0.35 | 0.012 | 0.28 | 0.01 | 0.22 | 0.009 | 0.18 | 0.008 | 0.14 | 0.007 |
| 2000 | 0.45 | 0.014 | 0.36 | 0.012 | 0.29 | 0.01 | 0.23 | 0.009 | 0.19 | 0.008 | 0.145 | 0.007 |
| 2500 | 0.46 | 0.013 | 0.37 | 0.012 | 0.3 | 0.01 | 0.24 | 0.009 | 0.19 | 0.008 | 0.15 | 0.007 |
| 3000 | 0.47 | 0.013 | 0.375 | 0.012 | 0.31 | 0.01 | 0.25 | 0.009 | 0.2 | 0.008 | 0.155 | 0.007 |
| 3500 | 0.48 | 0.013 | 0.38 | 0.012 | 0.32 | 0.01 | 0.255 | 0.009 | 0.205 | 0.008 | 0.16 | 0.007 |
| 4000 | 0.49 | 0.013 | 0.39 | 0.012 | 0.33 | 0.01 | 0.265 | 0.009 | 0.21 | 0.008 | 0.165 | 0.007 |
| 4500 | 0.495 | 0.013 | 0.395 | 0.012 | 0.335 | 0.01 | 0.275 | 0.009 | 0.22 | 0.008 | 0.175 | 0.007 |
| 5000 | 0.5 | 0.014 | 0.4 | 0.012 | 0.34 | 0.01 | 0.28 | 0.009 | 0.23 | 0.008 | 0.19 | 0.007 |

*Source: 10 N LOAD AA8011-5wt-TiB2-ECAP-COF- With-Error-Bars.xlsx. Error term is reproduced as provided in the source workbook.*

**Table S5. COF at 20 N: sliding-distance data (mean and error term)**

| **Sliding Distance (m)** | **Before ECAP Mean COF** | **Before ECAP Error ±** | **2 Passes ECAP Mean COF** | **2 Passes ECAP Error ±** | **4 Passes ECAP Mean COF** | **4 Passes ECAP Error ±** | **6 Passes ECAP Mean COF** | **6 Passes ECAP Error ±** | **8 Passes ECAP Mean COF** | **8 Passes ECAP Error ±** | **10 Passes ECAP Mean COF** | **10 Passes ECAP Error ±** |
| --- | --- | --- | --- | --- | --- | --- | --- | --- | --- | --- | --- | --- |
| 0 | 0.01 | 0.005 | 0.01 | 0.005 | 0.01 | 0.005 | 0.01 | 0.005 | 0.01 | 0.005 | 0.01 | 0.005 |
| 500 | 0.32 | 0.018 | 0.26 | 0.015 | 0.21 | 0.012 | 0.18 | 0.01 | 0.12 | 0.008 | 0.08 | 0.007 |
| 1000 | 0.42 | 0.014 | 0.34 | 0.012 | 0.27 | 0.01 | 0.2 | 0.009 | 0.14 | 0.007 | 0.1 | 0.006 |
| 1500 | 0.44 | 0.013 | 0.36 | 0.011 | 0.29 | 0.009 | 0.22 | 0.008 | 0.15 | 0.007 | 0.11 | 0.006 |
| 2000 | 0.45 | 0.013 | 0.37 | 0.011 | 0.3 | 0.009 | 0.23 | 0.008 | 0.16 | 0.007 | 0.115 | 0.006 |
| 2500 | 0.46 | 0.012 | 0.385 | 0.011 | 0.31 | 0.009 | 0.245 | 0.008 | 0.17 | 0.007 | 0.12 | 0.006 |
| 3000 | 0.47 | 0.012 | 0.395 | 0.011 | 0.32 | 0.009 | 0.255 | 0.008 | 0.18 | 0.007 | 0.125 | 0.006 |
| 3500 | 0.48 | 0.012 | 0.405 | 0.011 | 0.33 | 0.009 | 0.26 | 0.008 | 0.18 | 0.007 | 0.13 | 0.006 |
| 4000 | 0.49 | 0.013 | 0.42 | 0.011 | 0.34 | 0.009 | 0.265 | 0.008 | 0.19 | 0.007 | 0.14 | 0.006 |
| 4500 | 0.53 | 0.014 | 0.45 | 0.012 | 0.36 | 0.01 | 0.275 | 0.009 | 0.2 | 0.007 | 0.145 | 0.006 |
| 5000 | 0.62 | 0.016 | 0.5 | 0.013 | 0.38 | 0.01 | 0.29 | 0.009 | 0.21 | 0.008 | 0.16 | 0.007 |

*Source: 20 N LOAD AA8011-5wt-TiB2-ECAP-COF- With-Error-Bars.xlsx. Error term is reproduced as provided in the source workbook.*

**Table S6. COF at 30 N: sliding-distance data (mean and error term)**

| **Sliding Distance (m)** | **Before ECAP Mean COF** | **Before ECAP Error ±** | **2 Passes ECAP Mean COF** | **2 Passes ECAP Error ±** | **4 Passes ECAP Mean COF** | **4 Passes ECAP Error ±** | **6 Passes ECAP Mean COF** | **6 Passes ECAP Error ±** | **8 Passes ECAP Mean COF** | **8 Passes ECAP Error ±** | **10 Passes ECAP Mean COF** | **10 Passes ECAP Error ±** |
| --- | --- | --- | --- | --- | --- | --- | --- | --- | --- | --- | --- | --- |
| 0 | 0.01 | 0.005 | 0.01 | 0.005 | 0.01 | 0.005 | 0.01 | 0.005 | 0.01 | 0.005 | 0.01 | 0.005 |
| 500 | 0.31 | 0.018 | 0.24 | 0.015 | 0.2 | 0.012 | 0.16 | 0.01 | 0.12 | 0.008 | 0.08 | 0.007 |
| 1000 | 0.4 | 0.014 | 0.34 | 0.012 | 0.27 | 0.01 | 0.2 | 0.009 | 0.145 | 0.007 | 0.105 | 0.006 |
| 1500 | 0.43 | 0.013 | 0.36 | 0.011 | 0.29 | 0.009 | 0.215 | 0.008 | 0.16 | 0.007 | 0.12 | 0.006 |
| 2000 | 0.45 | 0.013 | 0.38 | 0.011 | 0.31 | 0.009 | 0.23 | 0.008 | 0.17 | 0.007 | 0.13 | 0.006 |
| 2500 | 0.47 | 0.012 | 0.395 | 0.011 | 0.32 | 0.009 | 0.245 | 0.008 | 0.18 | 0.007 | 0.14 | 0.006 |
| 3000 | 0.49 | 0.012 | 0.41 | 0.011 | 0.33 | 0.009 | 0.255 | 0.008 | 0.19 | 0.007 | 0.145 | 0.006 |
| 3500 | 0.5 | 0.012 | 0.42 | 0.011 | 0.34 | 0.009 | 0.265 | 0.008 | 0.2 | 0.007 | 0.15 | 0.006 |
| 4000 | 0.54 | 0.013 | 0.45 | 0.011 | 0.36 | 0.009 | 0.275 | 0.008 | 0.205 | 0.007 | 0.155 | 0.006 |
| 4500 | 0.59 | 0.014 | 0.48 | 0.012 | 0.39 | 0.01 | 0.29 | 0.009 | 0.215 | 0.007 | 0.16 | 0.006 |
| 5000 | 0.64 | 0.016 | 0.52 | 0.013 | 0.4 | 0.01 | 0.3 | 0.009 | 0.22 | 0.008 | 0.16 | 0.007 |

*Source: 30 N LOAD AA8011-5wt-TiB2-ECAP-COF- With-Error-Bars.xlsx. Error term is reproduced as provided in the source workbook.*

**Table S7. COF at 40 N: sliding-distance data (mean and error term)**

| **Sliding Distance (m)** | **Before ECAP Mean COF** | **Before ECAP Error ±** | **2 Passes ECAP Mean COF** | **2 Passes ECAP Error ±** | **4 Passes ECAP Mean COF** | **4 Passes ECAP Error ±** | **6 Passes ECAP Mean COF** | **6 Passes ECAP Error ±** | **8 Passes ECAP Mean COF** | **8 Passes ECAP Error ±** | **10 Passes ECAP Mean COF** | **10 Passes ECAP Error ±** |
| --- | --- | --- | --- | --- | --- | --- | --- | --- | --- | --- | --- | --- |
| 0 | 0.01 | 0.005 | 0.01 | 0.005 | 0.01 | 0.005 | 0.01 | 0.005 | 0.01 | 0.005 | 0.01 | 0.005 |
| 500 | 0.3 | 0.015 | 0.24 | 0.013 | 0.19 | 0.011 | 0.16 | 0.009 | 0.11 | 0.008 | 0.07 | 0.006 |
| 1000 | 0.39 | 0.013 | 0.33 | 0.011 | 0.26 | 0.009 | 0.2 | 0.008 | 0.14 | 0.007 | 0.1 | 0.006 |
| 1500 | 0.42 | 0.012 | 0.36 | 0.01 | 0.29 | 0.009 | 0.22 | 0.008 | 0.16 | 0.007 | 0.12 | 0.006 |
| 2000 | 0.44 | 0.012 | 0.38 | 0.01 | 0.31 | 0.009 | 0.24 | 0.008 | 0.17 | 0.007 | 0.13 | 0.006 |
| 2500 | 0.46 | 0.012 | 0.395 | 0.01 | 0.32 | 0.009 | 0.25 | 0.008 | 0.19 | 0.007 | 0.14 | 0.006 |
| 3000 | 0.48 | 0.012 | 0.41 | 0.01 | 0.33 | 0.009 | 0.26 | 0.008 | 0.2 | 0.007 | 0.15 | 0.006 |
| 3500 | 0.5 | 0.012 | 0.43 | 0.011 | 0.35 | 0.009 | 0.27 | 0.008 | 0.2 | 0.007 | 0.15 | 0.006 |
| 4000 | 0.53 | 0.013 | 0.45 | 0.011 | 0.37 | 0.009 | 0.29 | 0.008 | 0.21 | 0.007 | 0.16 | 0.006 |
| 4500 | 0.59 | 0.014 | 0.5 | 0.012 | 0.4 | 0.01 | 0.31 | 0.009 | 0.22 | 0.008 | 0.17 | 0.007 |
| 5000 | 0.65 | 0.016 | 0.54 | 0.013 | 0.41 | 0.01 | 0.31 | 0.009 | 0.23 | 0.008 | 0.17 | 0.007 |

*Source: 40 N LOAD AA8011-5wt-TiB2-ECAP-COF- With-Error-Bars.xlsx. Error term is reproduced as provided in the source workbook.*

**Table S8. COF at 50 N: sliding-distance data (mean and error term)**

| **Sliding Distance (m)** | **Before ECAP Mean COF** | **Before ECAP Error ±** | **2 Passes ECAP Mean COF** | **2 Passes ECAP Error ±** | **4 Passes ECAP Mean COF** | **4 Passes ECAP Error ±** | **6 Passes ECAP Mean COF** | **6 Passes ECAP Error ±** | **8 Passes ECAP Mean COF** | **8 Passes ECAP Error ±** | **10 Passes ECAP Mean COF** | **10 Passes ECAP Error ±** |
| --- | --- | --- | --- | --- | --- | --- | --- | --- | --- | --- | --- | --- |
| 0 | 0.01 | 0.005 | 0.01 | 0.005 | 0.01 | 0.005 | 0.01 | 0.005 | 0.01 | 0.005 | 0.01 | 0.005 |
| 500 | 0.3 | 0.015 | 0.23 | 0.013 | 0.18 | 0.011 | 0.15 | 0.009 | 0.1 | 0.008 | 0.075 | 0.006 |
| 1000 | 0.39 | 0.013 | 0.32 | 0.011 | 0.25 | 0.009 | 0.2 | 0.008 | 0.14 | 0.007 | 0.1 | 0.006 |
| 1500 | 0.42 | 0.012 | 0.35 | 0.01 | 0.28 | 0.009 | 0.22 | 0.008 | 0.16 | 0.007 | 0.115 | 0.006 |
| 2000 | 0.44 | 0.012 | 0.37 | 0.01 | 0.31 | 0.009 | 0.23 | 0.008 | 0.175 | 0.007 | 0.125 | 0.006 |
| 2500 | 0.47 | 0.012 | 0.39 | 0.01 | 0.32 | 0.009 | 0.245 | 0.008 | 0.185 | 0.007 | 0.13 | 0.006 |
| 3000 | 0.49 | 0.012 | 0.41 | 0.01 | 0.34 | 0.009 | 0.26 | 0.008 | 0.195 | 0.007 | 0.135 | 0.006 |
| 3500 | 0.51 | 0.012 | 0.42 | 0.011 | 0.35 | 0.009 | 0.27 | 0.008 | 0.2 | 0.007 | 0.14 | 0.006 |
| 4000 | 0.55 | 0.013 | 0.46 | 0.011 | 0.37 | 0.009 | 0.29 | 0.008 | 0.21 | 0.007 | 0.15 | 0.006 |
| 4500 | 0.6 | 0.014 | 0.5 | 0.012 | 0.4 | 0.01 | 0.3 | 0.009 | 0.22 | 0.008 | 0.16 | 0.007 |
| 5000 | 0.66 | 0.016 | 0.55 | 0.013 | 0.42 | 0.01 | 0.32 | 0.009 | 0.24 | 0.008 | 0.18 | 0.007 |

*Source: 50 N LAOD AA8011-5wt-TiB2-ECAP-COF- With-Error-Bars.xlsx. Error term is reproduced as provided in the source workbook.*

# Tables S9–S13. Wear-rate data

**Table S9. Wear rate at 10 N: sliding-distance data (mean and error term)**

| **Sliding Distance (m)** | **Before ECAP Mean (×10⁻⁶)** | **Before ECAP Error ± (×10⁻⁶)** | **2 Passes ECAP Mean (×10⁻⁶)** | **2 Passes ECAP Error ± (×10⁻⁶)** | **4 Passes ECAP Mean (×10⁻⁶)** | **4 Passes ECAP Error ± (×10⁻⁶)** | **6 Passes ECAP Mean (×10⁻⁶)** | **6 Passes ECAP Error ± (×10⁻⁶)** | **8 Passes ECAP Mean (×10⁻⁶)** | **8 Passes ECAP Error ± (×10⁻⁶)** | **10 Passes ECAP Mean (×10⁻⁶)** | **10 Passes ECAP Error ± (×10⁻⁶)** |
| --- | --- | --- | --- | --- | --- | --- | --- | --- | --- | --- | --- | --- |
| 0 | 2.1 | 0.15 | 1.7 | 0.12 | 1.32 | 0.1 | 1.05 | 0.08 | 0.82 | 0.07 | 0.65 | 0.06 |
| 500 | 1.5 | 0.09 | 1.23 | 0.08 | 1.02 | 0.07 | 0.82 | 0.06 | 0.62 | 0.05 | 0.48 | 0.05 |
| 1000 | 1.3 | 0.07 | 1.08 | 0.06 | 0.9 | 0.06 | 0.7 | 0.05 | 0.55 | 0.04 | 0.4 | 0.04 |
| 1500 | 1.18 | 0.06 | 0.98 | 0.05 | 0.82 | 0.05 | 0.64 | 0.04 | 0.49 | 0.04 | 0.36 | 0.03 |
| 2000 | 1.1 | 0.06 | 0.92 | 0.05 | 0.76 | 0.05 | 0.61 | 0.04 | 0.46 | 0.03 | 0.34 | 0.03 |
| 2500 | 1.04 | 0.05 | 0.88 | 0.05 | 0.72 | 0.04 | 0.58 | 0.04 | 0.43 | 0.03 | 0.31 | 0.03 |
| 3000 | 1.01 | 0.05 | 0.86 | 0.04 | 0.7 | 0.04 | 0.57 | 0.04 | 0.42 | 0.03 | 0.3 | 0.03 |
| 3500 | 0.98 | 0.05 | 0.84 | 0.04 | 0.69 | 0.04 | 0.55 | 0.04 | 0.41 | 0.03 | 0.29 | 0.03 |
| 4000 | 0.96 | 0.05 | 0.83 | 0.04 | 0.69 | 0.04 | 0.55 | 0.04 | 0.41 | 0.03 | 0.29 | 0.03 |
| 4500 | 0.96 | 0.05 | 0.83 | 0.04 | 0.7 | 0.04 | 0.57 | 0.04 | 0.43 | 0.03 | 0.31 | 0.03 |
| 5000 | 0.98 | 0.05 | 0.89 | 0.04 | 0.79 | 0.04 | 0.64 | 0.04 | 0.54 | 0.03 | 0.44 | 0.03 |

*Source: 10 N Load AA8011-5wt-TiB2-ECAP-Wear rate- With-Error-Bars.xlsx. The 50 N source dataset reports its final point at 5500 m; this value is reproduced without alteration.*

**Table S10. Wear rate at 20 N: sliding-distance data (mean and error term)**

| **Sliding Distance (m)** | **Before ECAP Mean** | **Before ECAP Error ±** | **2 Passes ECAP Mean** | **2 Passes ECAP Error ±** | **4 Passes ECAP Mean** | **4 Passes ECAP Error ±** | **6 Passes ECAP Mean** | **6 Passes ECAP Error ±** | **8 Passes ECAP Mean** | **8 Passes ECAP Error ±** | **10 Passes ECAP Mean** | **10 Passes ECAP Error ±** |
| --- | --- | --- | --- | --- | --- | --- | --- | --- | --- | --- | --- | --- |
| 0 | 2.9 | 0.22 | 2.38 | 0.18 | 1.88 | 0.15 | 1.43 | 0.12 | 1.15 | 0.1 | 0.78 | 0.08 |
| 500 | 2.18 | 0.14 | 1.76 | 0.12 | 1.43 | 0.1 | 1.08 | 0.08 | 0.76 | 0.07 | 0.52 | 0.06 |
| 1000 | 1.93 | 0.1 | 1.59 | 0.09 | 1.26 | 0.08 | 0.93 | 0.07 | 0.62 | 0.05 | 0.44 | 0.05 |
| 1500 | 1.81 | 0.09 | 1.49 | 0.08 | 1.18 | 0.07 | 0.87 | 0.06 | 0.59 | 0.05 | 0.39 | 0.04 |
| 2000 | 1.74 | 0.08 | 1.43 | 0.07 | 1.12 | 0.06 | 0.83 | 0.06 | 0.55 | 0.05 | 0.36 | 0.04 |
| 2500 | 1.68 | 0.08 | 1.39 | 0.07 | 1.09 | 0.06 | 0.81 | 0.05 | 0.54 | 0.04 | 0.35 | 0.04 |
| 3000 | 1.65 | 0.07 | 1.36 | 0.07 | 1.08 | 0.06 | 0.8 | 0.05 | 0.53 | 0.04 | 0.34 | 0.04 |
| 3500 | 1.63 | 0.07 | 1.35 | 0.06 | 1.07 | 0.06 | 0.8 | 0.05 | 0.53 | 0.04 | 0.34 | 0.03 |
| 4000 | 1.63 | 0.07 | 1.35 | 0.06 | 1.08 | 0.06 | 0.81 | 0.05 | 0.54 | 0.04 | 0.35 | 0.03 |
| 4500 | 1.66 | 0.07 | 1.39 | 0.07 | 1.13 | 0.06 | 0.85 | 0.06 | 0.59 | 0.05 | 0.4 | 0.04 |
| 5000 | 1.71 | 0.08 | 1.49 | 0.07 | 1.27 | 0.06 | 1.08 | 0.06 | 0.91 | 0.05 | 0.76 | 0.05 |

*Source: 20 N Load AA8011-5wt-TiB2-ECAP-Wear rate- With-Error-Bars.xlsx. The 50 N source dataset reports its final point at 5500 m; this value is reproduced without alteration.*

**Table S11. Wear rate at 30 N: sliding-distance data (mean and error term)**

| **Sliding Distance (m)** | **Before ECAP Mean** | **Before ECAP Error ±** | **2 Passes ECAP Mean** | **2 Passes ECAP Error ±** | **4 Passes ECAP Mean** | **4 Passes ECAP Error ±** | **6 Passes ECAP Mean** | **6 Passes ECAP Error ±** | **8 Passes ECAP Mean** | **8 Passes ECAP Error ±** | **10 Passes ECAP Mean** | **10 Passes ECAP Error ±** |
| --- | --- | --- | --- | --- | --- | --- | --- | --- | --- | --- | --- | --- |
| 0 | 4.4 | 0.28 | 3.7 | 0.24 | 2.8 | 0.2 | 1.95 | 0.15 | 1.35 | 0.12 | 1.08 | 0.1 |
| 500 | 3.25 | 0.18 | 2.65 | 0.15 | 1.9 | 0.12 | 1.35 | 0.1 | 0.92 | 0.08 | 0.8 | 0.07 |
| 1000 | 2.75 | 0.14 | 2.15 | 0.12 | 1.6 | 0.1 | 1.15 | 0.08 | 0.72 | 0.07 | 0.6 | 0.06 |
| 1500 | 2.4 | 0.12 | 1.9 | 0.1 | 1.4 | 0.09 | 1 | 0.07 | 0.62 | 0.06 | 0.52 | 0.05 |
| 2000 | 2.2 | 0.11 | 1.72 | 0.09 | 1.25 | 0.08 | 0.95 | 0.07 | 0.58 | 0.05 | 0.46 | 0.05 |
| 2500 | 2.1 | 0.1 | 1.63 | 0.09 | 1.15 | 0.08 | 0.92 | 0.06 | 0.55 | 0.05 | 0.41 | 0.04 |
| 3000 | 2 | 0.1 | 1.58 | 0.08 | 1.1 | 0.07 | 0.9 | 0.06 | 0.52 | 0.05 | 0.38 | 0.04 |
| 3500 | 1.98 | 0.1 | 1.59 | 0.08 | 1.08 | 0.07 | 0.9 | 0.06 | 0.52 | 0.05 | 0.38 | 0.04 |
| 4000 | 2.05 | 0.1 | 1.62 | 0.08 | 1.12 | 0.07 | 0.92 | 0.06 | 0.54 | 0.05 | 0.4 | 0.04 |
| 4500 | 2.15 | 0.11 | 1.72 | 0.09 | 1.22 | 0.08 | 1 | 0.07 | 0.62 | 0.06 | 0.48 | 0.05 |
| 5000 | 2.52 | 0.12 | 2.19 | 0.11 | 1.86 | 0.1 | 1.58 | 0.08 | 1.31 | 0.07 | 1.09 | 0.06 |

*Source: 30 N Load AA8011-5wt-TiB2-ECAP-Wear rate- With-Error-Bars.xlsx. The 50 N source dataset reports its final point at 5500 m; this value is reproduced without alteration.*

**Table S12. Wear rate at 40 N: sliding-distance data (mean and error term)**

| **Sliding Distance (m)** | **Before ECAP Mean** | **Before ECAP Error ±** | **2 Passes ECAP Mean** | **2 Passes ECAP Error ±** | **4 Passes ECAP Mean** | **4 Passes ECAP Error ±** | **6 Passes ECAP Mean** | **6 Passes ECAP Error ±** | **8 Passes ECAP Mean** | **8 Passes ECAP Error ±** | **10 Passes ECAP Mean** | **10 Passes ECAP Error ±** |
| --- | --- | --- | --- | --- | --- | --- | --- | --- | --- | --- | --- | --- |
| 0 | 5.7 | 0.3 | 4.6 | 0.25 | 3.6 | 0.22 | 2.5 | 0.18 | 2 | 0.15 | 1.5 | 0.12 |
| 500 | 4.65 | 0.22 | 3.6 | 0.18 | 2.8 | 0.15 | 1.95 | 0.13 | 1.4 | 0.1 | 1.05 | 0.09 |
| 1000 | 4.1 | 0.18 | 3.1 | 0.15 | 2.3 | 0.13 | 1.55 | 0.1 | 1.1 | 0.09 | 0.8 | 0.07 |
| 1500 | 3.85 | 0.16 | 2.7 | 0.13 | 2 | 0.11 | 1.3 | 0.09 | 0.95 | 0.08 | 0.65 | 0.06 |
| 2000 | 3.7 | 0.15 | 2.65 | 0.12 | 1.95 | 0.1 | 1.25 | 0.09 | 0.9 | 0.07 | 0.6 | 0.06 |
| 2500 | 3.5 | 0.14 | 2.5 | 0.12 | 1.8 | 0.1 | 1.1 | 0.08 | 0.8 | 0.07 | 0.55 | 0.05 |
| 3000 | 3.4 | 0.14 | 2.4 | 0.11 | 1.7 | 0.09 | 1 | 0.08 | 0.75 | 0.06 | 0.52 | 0.05 |
| 3500 | 3.3 | 0.14 | 2.35 | 0.11 | 1.65 | 0.09 | 0.98 | 0.07 | 0.72 | 0.06 | 0.5 | 0.05 |
| 4000 | 3.25 | 0.14 | 2.32 | 0.11 | 1.65 | 0.09 | 0.97 | 0.07 | 0.72 | 0.06 | 0.5 | 0.05 |
| 4500 | 3.3 | 0.15 | 2.5 | 0.12 | 1.9 | 0.1 | 1.2 | 0.08 | 1.05 | 0.08 | 0.9 | 0.07 |
| 5000 | 3.36 | 0.16 | 2.9 | 0.14 | 2.45 | 0.12 | 1.8 | 0.1 | 1.71 | 0.09 | 1.43 | 0.08 |

*Source: 40 N Load AA8011-5wt-TiB2-ECAP-Wear rate- With-Error-Bars.xlsx. The 50 N source dataset reports its final point at 5500 m; this value is reproduced without alteration.*

**Table S13. Wear rate at 50 N: sliding-distance data (mean and error term)**

| **Sliding Distance (m)** | **Before ECAP Mean** | **Before ECAP Error ±** | **2 Passes ECAP Mean** | **2 Passes ECAP Error ±** | **4 Passes ECAP Mean** | **4 Passes ECAP Error ±** | **6 Passes ECAP Mean** | **6 Passes ECAP Error ±** | **8 Passes ECAP Mean** | **8 Passes ECAP Error ±** | **10 Passes ECAP Mean** | **10 Passes ECAP Error ±** |
| --- | --- | --- | --- | --- | --- | --- | --- | --- | --- | --- | --- | --- |
| 0 | 7.2 | 0.45 | 5.5 | 0.35 | 4.2 | 0.3 | 3.2 | 0.25 | 2.3 | 0.2 | 1.65 | 0.15 |
| 1000 | 4.55 | 0.25 | 3.8 | 0.22 | 2.9 | 0.18 | 2.2 | 0.15 | 1.55 | 0.12 | 1.1 | 0.1 |
| 2000 | 3.65 | 0.2 | 3 | 0.18 | 2.3 | 0.15 | 1.65 | 0.12 | 1.15 | 0.1 | 0.8 | 0.08 |
| 3000 | 3.1 | 0.18 | 2.65 | 0.16 | 2 | 0.13 | 1.45 | 0.11 | 0.95 | 0.09 | 0.65 | 0.07 |
| 4000 | 2.9 | 0.18 | 2.45 | 0.15 | 1.85 | 0.12 | 1.35 | 0.1 | 0.85 | 0.08 | 0.55 | 0.06 |
| 5500 | 4.2 | 0.22 | 3.45 | 0.18 | 2.65 | 0.15 | 2.02 | 0.12 | 1.31 | 0.1 | 1.16 | 0.09 |

*Source: 50 N Load AA8011-5wt-TiB2-ECAP-Wear rate- With-Error-Bars.xlsx. The 50 N source dataset reports its final point at 5500 m; this value is reproduced without alteration.*

# Tables S14–S19. Processing-map power-dissipation efficiency data

**Table S14. Power-dissipation efficiency matrix: Before ECAP**

| **Temperature (°C)** | **1e-03** | **3e-03** | **1e-02** | **3e-02** | **1e-01** | **3e-01** | **1e+00** | **3e+00** | **1e+01** | **3e+01** | **1e+02** |
| --- | --- | --- | --- | --- | --- | --- | --- | --- | --- | --- | --- |
| 250 | 5.1 | 10.2 | 17.6 | 23.8 | 26.7 | 24.3 | 17.6 | 10.8 | 5.1 | 2.1 | 0.6 |
| 275 | 8.8 | 17.6 | 30.5 | 41.2 | 46.2 | 42.1 | 30.5 | 18.7 | 8.8 | 3.6 | 1.1 |
| 300 | 13.1 | 26.3 | 45.6 | 61.6 | 69.1 | 62.9 | 45.7 | 28 | 13.2 | 5.5 | 1.7 |
| 325 | 17 | 34 | 58.8 | 79.6 | 89.2 | 81.2 | 59.1 | 36.3 | 17.2 | 7.2 | 2.3 |
| 350 | 19 | 38 | 65.7 | 88.8 | 99.5 | 90.7 | 66.1 | 40.7 | 19.4 | 8.2 | 2.6 |
| 375 | 18.4 | 36.7 | 63.4 | 85.6 | 96 | 87.6 | 64 | 39.5 | 19 | 8.1 | 2.6 |
| 400 | 15.4 | 30.7 | 52.8 | 71.4 | 80.1 | 73.1 | 53.5 | 33.2 | 16 | 6.9 | 2.3 |
| 425 | 11.1 | 22.1 | 38.1 | 51.4 | 57.7 | 52.8 | 38.7 | 24.1 | 11.8 | 5.1 | 1.8 |
| 450 | 7 | 13.8 | 23.7 | 32 | 35.9 | 32.9 | 24.2 | 15.2 | 7.4 | 3.3 | 1.1 |
| 475 | 3.8 | 7.4 | 12.7 | 17.1 | 19.2 | 17.6 | 13 | 8.2 | 4.1 | 1.8 | 0.6 |
| 500 | 1.7 | 3.4 | 5.9 | 7.9 | 8.9 | 8.2 | 6 | 3.8 | 1.9 | 0.9 | 0.3 |

*Source: BEFORE ECAP AA8011-5wt-TiB2-ECAP-Processing-Map-Eta-Data.xlsx. η values are given in percent and correspond to the temperature/strain-rate grid used for the processing maps.*

**Table S15. Power-dissipation efficiency matrix: 2 passes**

| **Temperature (°C)** | **1e-04** | **1e-03** | **1e-02** | **1e-01** | **1e+00** | **1e+01** | **1e+02** |
| --- | --- | --- | --- | --- | --- | --- | --- |
| 250 | 10 | 20 | 30 | 45 | 55 | 35 | 10 |
| 275 | 15 | 30 | 45 | 65 | 75 | 55 | 20 |
| 300 | 25 | 40 | 60 | 85 | 95 | 70 | 35 |
| 325 | 35 | 55 | 75 | 95 | 100 | 80 | 50 |
| 350 | 40 | 65 | 85 | 98 | 95 | 75 | 45 |
| 375 | 45 | 70 | 90 | 95 | 85 | 65 | 35 |
| 400 | 50 | 65 | 80 | 85 | 75 | 55 | 25 |
| 425 | 50 | 60 | 70 | 75 | 60 | 40 | 20 |
| 450 | 45 | 55 | 60 | 65 | 45 | 30 | 15 |
| 475 | 35 | 45 | 50 | 50 | 35 | 20 | 10 |
| 500 | 25 | 35 | 40 | 40 | 25 | 15 | 5 |

*Source: 2 PASSES ECAP AA8011-5wt-TiB2-ECAP-Processing-Map-Eta-Data.xlsx. η values are given in percent and correspond to the temperature/strain-rate grid used for the processing maps.*

**Table S16. Power-dissipation efficiency matrix: 4 passes**

| **Temperature (°C)** | **1e-04** | **1e-03** | **1e-02** | **1e-01** | **1e+00** | **1e+01** | **1e+02** |
| --- | --- | --- | --- | --- | --- | --- | --- |
| 250 | 8 | 15 | 28 | 42 | 55 | 45 | 20 |
| 275 | 12 | 25 | 45 | 65 | 78 | 60 | 30 |
| 300 | 20 | 40 | 68 | 88 | 100 | 78 | 42 |
| 325 | 30 | 58 | 82 | 98 | 100 | 88 | 55 |
| 350 | 40 | 70 | 90 | 100 | 98 | 82 | 62 |
| 375 | 48 | 78 | 98 | 100 | 95 | 75 | 55 |
| 400 | 55 | 82 | 95 | 92 | 82 | 62 | 45 |
| 425 | 58 | 78 | 85 | 78 | 65 | 48 | 32 |
| 450 | 55 | 70 | 72 | 65 | 52 | 35 | 22 |
| 475 | 45 | 58 | 60 | 52 | 40 | 28 | 15 |
| 500 | 32 | 45 | 48 | 40 | 30 | 20 | 10 |

*Source: 4 PASSES ECAP AA8011-5wt-TiB2-ECAP-Processing-Map-Eta-Data.xlsx. η values are given in percent and correspond to the temperature/strain-rate grid used for the processing maps.*

**Table S17. Power-dissipation efficiency matrix: 6 passes**

| **Temperature (°C)** | **1e-04** | **1e-03** | **1e-02** | **1e-01** | **1e+00** | **1e+01** | **1e+02** |
| --- | --- | --- | --- | --- | --- | --- | --- |
| 250 | 8 | 15 | 28 | 42 | 55 | 45 | 25 |
| 275 | 12 | 25 | 42 | 60 | 72 | 58 | 30 |
| 300 | 20 | 38 | 58 | 78 | 92 | 70 | 40 |
| 325 | 28 | 52 | 72 | 92 | 100 | 82 | 50 |
| 350 | 38 | 68 | 88 | 98 | 96 | 78 | 55 |
| 375 | 48 | 78 | 96 | 100 | 94 | 72 | 48 |
| 400 | 55 | 80 | 92 | 90 | 78 | 58 | 38 |
| 425 | 58 | 72 | 82 | 78 | 65 | 45 | 28 |
| 450 | 55 | 65 | 70 | 65 | 52 | 35 | 20 |
| 475 | 45 | 55 | 58 | 50 | 38 | 25 | 12 |
| 500 | 30 | 42 | 45 | 38 | 28 | 18 | 8 |

*Source: 6 PASSES ECAP AA8011-5wt-TiB2-ECAP-Processing-Map-Eta-Data.xlsx. η values are given in percent and correspond to the temperature/strain-rate grid used for the processing maps.*

**Table S18. Power-dissipation efficiency matrix: 8 passes**

| **Temperature (°C)** | **1e-04** | **1e-03** | **1e-02** | **1e-01** | **1e+00** | **1e+01** | **1e+02** |
| --- | --- | --- | --- | --- | --- | --- | --- |
| 250 | 8 | 15 | 25 | 38 | 45 | 25 | 8 |
| 275 | 12 | 25 | 42 | 58 | 68 | 45 | 15 |
| 300 | 20 | 38 | 60 | 78 | 88 | 60 | 25 |
| 325 | 30 | 55 | 78 | 95 | 98 | 78 | 38 |
| 350 | 42 | 70 | 88 | 100 | 98 | 85 | 48 |
| 375 | 52 | 80 | 96 | 100 | 92 | 72 | 40 |
| 400 | 58 | 82 | 92 | 90 | 78 | 58 | 30 |
| 425 | 55 | 75 | 82 | 78 | 65 | 45 | 22 |
| 450 | 48 | 65 | 68 | 62 | 52 | 35 | 15 |
| 475 | 38 | 52 | 55 | 48 | 38 | 25 | 10 |
| 500 | 25 | 40 | 42 | 35 | 28 | 18 | 7 |

*Source: 8 ECAP PASSES AA8011-5wt-TiB2-ECAP-Processing-Map-Eta-Data.xlsx. η values are given in percent and correspond to the temperature/strain-rate grid used for the processing maps.*

**Table S19. Power-dissipation efficiency matrix: 10 passes**

| **Temperature (°C)** | **1e-04** | **1e-03** | **1e-02** | **1e-01** | **1e+00** | **1e+01** | **1e+02** |
| --- | --- | --- | --- | --- | --- | --- | --- |
| 250 | 8 | 15 | 25 | 38 | 45 | 25 | 8 |
| 275 | 12 | 25 | 42 | 58 | 68 | 45 | 15 |
| 300 | 20 | 38 | 60 | 78 | 88 | 60 | 25 |
| 325 | 30 | 55 | 75 | 92 | 98 | 78 | 38 |
| 350 | 42 | 70 | 88 | 100 | 98 | 85 | 48 |
| 375 | 52 | 80 | 96 | 100 | 92 | 72 | 40 |
| 400 | 58 | 82 | 92 | 90 | 78 | 58 | 30 |
| 425 | 55 | 75 | 82 | 78 | 65 | 45 | 22 |
| 450 | 48 | 65 | 68 | 62 | 52 | 35 | 15 |
| 475 | 38 | 52 | 55 | 48 | 38 | 25 | 10 |
| 500 | 25 | 40 | 42 | 35 | 28 | 18 | 7 |

*Source: 10 ECAP PASSES AA8011-5wt-TiB2-ECAP-Processing-Map-Eta-Data.xlsx. η values are given in percent and correspond to the temperature/strain-rate grid used for the processing maps.*

# Table S20. Wear-height-loss summary at 5000 m

| **Normal load** | **ECAP condition** | **Sliding distance (m)** | **Wear-height loss (µm)** | **Error ± (µm)** |
| --- | --- | --- | --- | --- |
| 20 N | Before ECAP | 5000 | 820 | 16 |
| 20 N | 2 Passes ECAP | 5000 | 740 | 15 |
| 20 N | 4 Passes ECAP | 5000 | 660 | 14 |
| 20 N | 6 Passes ECAP | 5000 | 590 | 13 |
| 20 N | 8 Passes ECAP | 5000 | 520 | 12 |
| 20 N | 10 Passes ECAP | 5000 | 470 | 11 |
| 30 N | Before ECAP | 5000 | 1040 | 22 |
| 30 N | 2 Passes ECAP | 5000 | 940 | 20 |
| 30 N | 4 Passes ECAP | 5000 | 850 | 18 |
| 30 N | 6 Passes ECAP | 5000 | 760 | 17 |
| 30 N | 8 Passes ECAP | 5000 | 680 | 16 |
| 30 N | 10 Passes ECAP | 5000 | 610 | 15 |
| 40 N | Before ECAP | 5000 | 1260 | 26 |
| 40 N | 2 Passes ECAP | 5000 | 1140 | 24 |
| 40 N | 4 Passes ECAP | 5000 | 1030 | 22 |
| 40 N | 6 Passes ECAP | 5000 | 920 | 21 |
| 40 N | 8 Passes ECAP | 5000 | 830 | 20 |
| 40 N | 10 Passes ECAP | 5000 | 740 | 19 |
| 50 N | Before ECAP | 5000 | 1500 | 30 |
| 50 N | 2 Passes ECAP | 5000 | 1350 | 27 |
| 50 N | 4 Passes ECAP | 5000 | 1220 | 25 |
| 50 N | 6 Passes ECAP | 5000 | 1100 | 23 |
| 50 N | 8 Passes ECAP | 5000 | 980 | 22 |
| 50 N | 10 Passes ECAP | 5000 | 880 | 21 |

*Source: the five wear-height-loss workbooks. This compact table provides the 5000 m summary points; the detailed source workbooks contain the full sliding-distance series.*

# Notes on data presentation

The Supporting Information is limited to processed numerical data directly associated with the quantitative results and figures in the manuscript; raw instrument files, laboratory notebooks, and unrelated intermediate records are not included.

Where the source workbooks identify an error term as a visual estimate, that wording has not been changed in this Supporting Information.

No values have been added from external sources or inferred to complete missing measurements. Source workbook values are reproduced as supplied.

For the 50 N wear-rate dataset, the source workbook reports the final point at 5500 m rather than 5000 m; the supplied value is retained as reported.
